# The InBIO Barcoding Initiative Database: DNA barcodes of Orthoptera from Portugal

**DOI:** 10.3897/BDJ.12.e118010

**Published:** 2024-05-15

**Authors:** Sílvia Pina, Joana Pauperio, Francisco Barros, Cátia Chaves, Filipa MS Martins, Joana Pinto, Joana Veríssimo, Vanessa A Mata, Pedro Beja, Sónia Ferreira

**Affiliations:** 1 CIBIO, Centro de Investigação em Biodiversidade e Recursos Genéticos, InBIO Laboratório Associado, Campus de Vairão, Universidade do Porto, 4485–661 Vairão, Vila do Conde, Portugal CIBIO, Centro de Investigação em Biodiversidade e Recursos Genéticos, InBIO Laboratório Associado, Campus de Vairão, Universidade do Porto, 4485–661 Vairão Vila do Conde Portugal; 2 BIOPOLIS Program in Genomics, Biodiversity and Land Planning, CIBIO, Campus de Vairão, 4485–661 Vairão, Vila do Conde, Portugal BIOPOLIS Program in Genomics, Biodiversity and Land Planning, CIBIO, Campus de Vairão, 4485–661 Vairão Vila do Conde Portugal; 3 CIBIO, Centro de Investigação em Biodiversidade e Recursos Genéticos, InBIO Laboratório Associado, Instituto Superior de Agronomia, Universidade de Lisboa, Lisboa, Portugal CIBIO, Centro de Investigação em Biodiversidade e Recursos Genéticos, InBIO Laboratório Associado, Instituto Superior de Agronomia, Universidade de Lisboa Lisboa Portugal; 4 European Molecular Biology Laboratory, European Bioinformatics Institute, Wellcome Genome Campus, Hinxton, Cambridge, United Kingdom European Molecular Biology Laboratory, European Bioinformatics Institute Wellcome Genome Campus, Hinxton, Cambridge United Kingdom; 5 Rua da Eira 3, S. Salvador, 2550-251, Cercal, Portugal Rua da Eira 3, S. Salvador, 2550-251 Cercal Portugal; 6 EBM, Estação Biológica de Mertola, Praça Luis de Camoes, Mertola, Mertola, Portugal EBM, Estação Biológica de Mertola, Praça Luis de Camoes, Mertola Mertola Portugal

**Keywords:** Orthoptera, species distributions, continental Portugal, DNA barcode, cytochrome c oxidase subunit I (COI)

## Abstract

**Background:**

The InBIO Barcoding Initiative (IBI) Orthoptera dataset contains records of 420 specimens covering all the eleven Orthoptera families occurring in Portugal. Specimens were collected in continental Portugal from 2005 to 2021 and were morphologically identified to species level by taxonomists. A total of 119 species were identified corresponding to about 77% of all the orthopteran species known from continental Portugal.

**New information:**

DNA barcodes of 54 taxa were made public for the first time at the Barcode of Life Data System (BOLD). Furthermore, the submitted sequences were found to cluster in 129 BINs (Barcode Index Numbers), 35 of which were new additions to the Barcode of Life Data System (BOLD). All specimens have their DNA barcodes publicly accessible through BOLD online database. *Stenobothruslineatus* is recorded for the first time for continental Portugal. This dataset greatly increases the knowledge on the DNA barcodes and distribution of Orthoptera from Portugal. All DNA extractions and most specimens are deposited in the IBI collection at CIBIO, Research Center in Biodiversity and Genetic Resources.

## Introduction

Insects are very challenging to study due to their astonishing biological diversity, the lack of taxonomic expertise for several groups and traditional morphology-based species identification being often logistically or financially unsustainable. DNA metabarcoding is rapidly emerging to overcome these challenges (van Klink et al. 2022), but its application relies on the existence of comprehensive reference collections of DNA barcodes. This method identifies multiple species from a mixed sample, based on DNA barcoding using high-throughput sequencing (HTS) of a specific DNA marker, usually the mitochondrial cytochrome c oxidase I (COI) gene. DNA barcoding is a molecular biology method for species identification that relies on the comparison of a short mitochondrial DNA sequence of interest to a library of sequences with known species identity (Hebert et al. 2003). Hence, for these comparisons, it is important to guarantee that a good sequence library is being used, i.e. all DNA barcodes come from specimens identified by taxonomists (Chimeno et al. 2023). The Barcode of Life Data Systems (BOLD) is an international barcode reference database made available in 2007 (Ratnasingham and Herbert 2007) and large national barcoding initiatives worldwide have contributed to increasing this library (Janzen and Hallwachs 2011, Geiger et al. 2016, deWaard et al. 2019). In Portugal, the InBIO Barcoding Initiative (IBI) was the first project aimed to develop a reference collection of DNA barcoding sequences, having largely focused on Portuguese invertebrate taxa, particularly insects (Ferreira et al. 2018).

Orthoptera are a diverse group of herbivorous insects and playing an important role in ecosystem functioning as both primary consumers in different habitats and significant food sources for higher trophic levels (e.g. birds, mammals) (Catry et al. 2018, Valdez and Cryan 2013). Due to the strong connection with plants, orthopterans are good indicators of habitat changes and, therefore, often used in environmental monitoring and assessment (Bieringer et al. 2013, Vasconcelos et al. 2019). However, despite their functional importance, they are still poorly studied in areas with high levels of endemic species, such as the Iberian Peninsula (Hochkirch et al. 2016). Studies on the Portuguese Orthoptera fauna have been published in a very scattered manner over time, generally lacking comprehensive inventories or focusing on certain Orthoptera taxa (Pina et al. 2017). Although some of these more recent studies report new findings for the country (e.g. Lemos et al. (2016), Monteiro et al. (2016)), the prime information, such as which species occur in Portugal and their distributions, remains very incomplete.

In Europe, large DNA barcoding initiatives have been established mainly in the northern and central countries (Gaytán et al. 2020). In the study conducted by Hawlitschek et al. (2017), DNA barcodes of Orthoptera from four barcoding initiatives (Barcoding Fauna Bavarica (Germany), German Barcode of Life, Austrian Barcode of Life and Swiss Barcode of Life) comprising three central European countries (Austria, Germany and Switzerland) were made available. A total of 748 COI sequences were obtained for several central European Orthoptera taxa that also occur in other countries, namely in Portugal. This study also showed that barcoding studies can be successfully applied to Orthoptera revealing an overall congruence of 76.2% of the 127 Orthoptera taxa in the study. However, more work is necessary to create a library of barcodes of the European Orthoptera because the representation of certain groups (e.g. Tetrigidae, Gryllidae and Bradyporinae) (Kasalo et al. 2023) and regional areas, such as the southern European peninsulas, remains insufficient. The Iberian Peninsula is a known hotspot of endemic Orthoptera species, but many of its species remain unrepresented in the DNA barcoding reference collections.

The IBI Orthoptera dataset contains records of 420 specimens of Orthoptera collected in continental Portugal, all morphologically identified to species level, for a total of 119 species. Our results constitute a first step in the construction of a DNA barcode database of a curated reference collection of Iberian Orthoptera species.

## General description

### Purpose

This dataset aims to provide a contribution to the knowledge on DNA barcodes of Portuguese Orthoptera. Such a library should facilitate DNA-based identification of species for both traditional molecular studies and DNA metabarcoding studies. Furthermore, it constitutes a valuable resource for taxonomic research on Iberian Orthoptera and their distribution.

### Additional information

A total of 420 specimens of orthopterans were collected and DNA barcoded (Table 1, Suppl. material 2) corresponding to 119 species, about 77% of all the orthopteran species known from continental Portugal (Aires and Menano 1915, Pina et al. 2017, GBIF 2023, IUCN 2023). Figs 1, 2 illustrate examples of the diversity of species that are part of the dataset of distribution data and DNA barcodes of Portuguese Orthoptera. The dataset includes 39 Iberian endemic species, of which six occur only in continental Portugal: *Ephippigeridarosae*, *Lluciapomaresiusanapaulae*, *Neocallicraniabarrosi*, *Neocallicraniaserrata*, *Pterolepislusitanica* and *Pycnogastercucullatus* (Table 1). Additionally, the species *Stenobothruslineatus* is recorded for the first time for Portugal in this dataset. A full-length barcode of 658 bp was obtained for all 420 specimens. This dataset contributes significantly to the representation of both species and genetic diversity of Orthoptera in public libraries. Of the 120 taxa barcoded, DNA barcodes of 54 are made public for the first time (marked with # in Taxa field of Table 1). The submitted sequences were found to cluster in 129 BINs, 35 of which were new to BOLD (unique BINs, marked with " in Taxa field of Table 1).

The results show multiple BINs for a recognised species in several cases. Namely, three BINs were obtained in each of the recognised species given: *Antaxiusspinibrachius*, *Nemobiussylvestris*, *Tessellanatessellata* and *Thyreonotusbidens*. Two BINs were obtained in each of the following taxa: *Gryllomorphalongicauda*, *Gryllotalpavineae*, *Lluciapomaresiusasturiensis*, *Neocallicranialusitanica*, *Pezotettixgiornae*, *Pycnogastercucullatus*, *Platycleissabulosa* and for the subspecies *Neocallicraniaselligerameridionalis*. Our dataset includes three BINs obtained for the species *Antaxiusspinibrachius*, including the first sequences of the BIN BOLD:AER0568 from specimens collected in the Castelo Branco and Guarda Districts. In a previous study, aimed at the phylogeography of this species in the Iberian Peninsula, two different lineages of this species were identified, one of which occurs along the Cordillera Oretana and the other includes all other populations (Gutiérrez-Rodríguez et al. 2014). The genetic diversity found in DNA barcodes in this species highlights the necessity of a taxonomic revision. Additionally, in the family Tettigoniidae, the specimen of the *Neocallicrania* genus collected in the Setúbal Peninsula was morphologically identified as *Neocallicranialusitanica* using the available literature (Barat 2007) and the unique BIN BOLD:AEO6978 was obtained. However, the revision conducted by Barat (2013) pointed out uncertainties related to specimens collected in this geographic area. Thus, our results emphasise the need for further work towards a better understanding of the taxonomy of this genus in the Iberian Peninsula.

Of the 119 species, 104 have direct correspondence between morphologic identification and BINs, leaving 15 species involved in BIN sharing. In the subfamily Tettigoniinae, two BINs were found to be shared by more than one species, the BIN BOLD:AEO6325 and the BIN BOLD:AEO7101 was shared by *Platycleisalbopunctata* and *Platycleissabulosa* and by *Pterolepislusitanica* and *Pterolepisspoliata*, respectively. In the subfamily Gomphocerinae, the generated sequence of *Pseudochorthippusparallelus* shared BIN BOLD:AAC3399 with sequences of *Pseudochorthippusmontanus* from Germany, Ukraine and Norway. Introgression caused by hybridisation is a well-studied phenomenon in areas where both species occur in sympatry in Germany (Hochkirch and Lemke 2011, Rohde et al. 2015). These species are morphologically very similar, but have different ecological requirements and both species occur in Spain, although *Pseudochorthippusmontanus* is restricted to the northeast (Llucià-Pomares 2002). Additionally, in this subfamily, there are other Gomphocerinae groups of species that cannot be identified using the DNA barcode COI fragment. Sequences obtained from our specimens of *Chorthippusbinotatus*, *Chorthippusjacobsi* and *Chorthippusyersini* shared the same BIN BOLD:AAC5779. Moreover and in line with the results by Hawlitschek et al. (2017), sequences obtained as *Omocestusrufipes* and *Omocestusviridulus* shared the same BIN. Likewise, in the subfamily Oedipodinae, two BINs were shared by more than one species, namely the BIN BOLD:AAJ3344 was shared by *Sphingonotusazurescens* and *Sphingonotusnodulosus* and the BIN BOLD:AEI2886 was shared by *Sphingonotuslluciapomaresi* and *Sphingonotuslusitanicus*. The success rate of DNA barcoding in the genus *Sphingonotus* was previously studied by Dey et al. (2020). Their results showed intensive sharing of identical or closely-related DNA barcodes indicating that the single analysis of the COI fragment is not sufficient enough to differentiate many of the *Sphingonotus* species.

This study shows that DNA barcode sequences, based on the COI mitochondrial gene fragment, can be useful in identifying Portuguese Orthoptera to species level. To our knowledge, this is the first study to focus on DNA barcoding of the Orthoptera order for the Iberian Peninsula. It also highlights several taxonomic challenges related to the rich fauna of this group and suggests intricated phylogeographic processes in the region that lead to the diversification of several taxa and high endemism levels in line with what previous studies have found (Barranco 2004, Solé et al. 2018). Our results constitute a first step in the construction of a DNA barcode database of a curated reference collection of Iberian (Portuguese) Orthoptera species that can be used in studies where it is necessary to identify specimens either by DNA barcoding or DNA metabarcoding.

## Project description

### Title

The InBIO Barcoding Initiative Database: DNA barcodes of Orthoptera from Portugal.

### Personnel

Pedro Beja (project coordinator), Sónia Ferreira (taxonomist and IBI manager), Sílvia Pina (taxonomist), Joana Paupério (IBI manager), Francisco Barros (taxonomist), Cátia Chaves (project technician), Filipa M.S. Martins (molecular biologist), Joana Pinto, Joana Veríssimo (molecular biologist), Vanessa Mata (molecular biologist).

### Study area description

**Figure 3.**
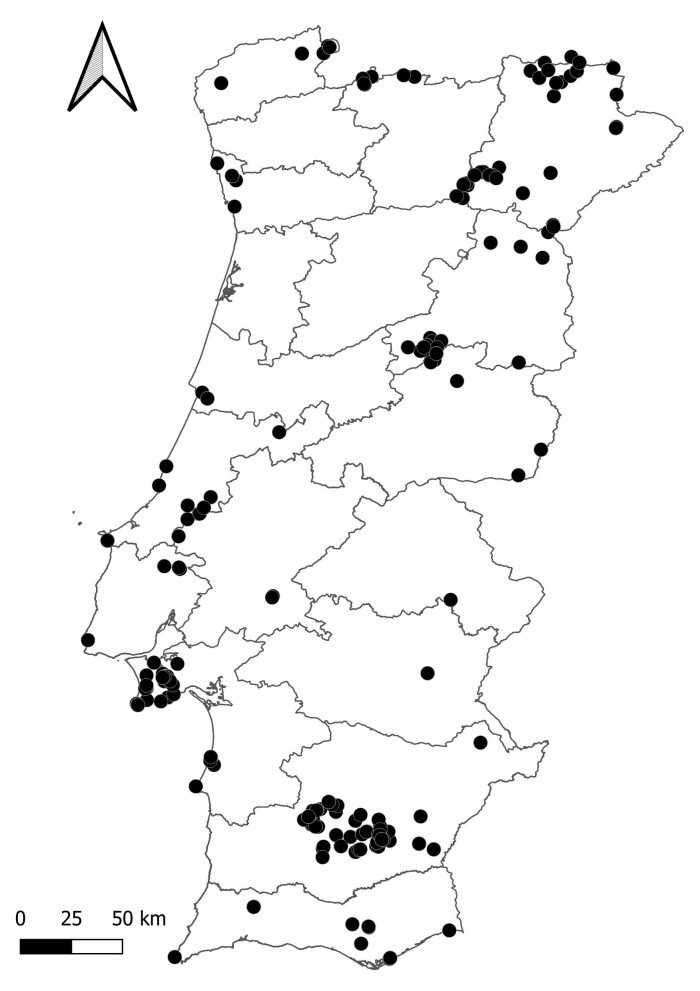
Map of the localities where Orthoptera samples were collected in continental Portugal. Portuguese districts are also represented.

Continental Portugal (Fig. 3).

### Design description

Orthoptera specimens were collected in the field, morphologically identified and DNA barcoded.

### Funding

The present work was funded by National Funds through FCT-Fundação para a Ciência e a Tecnologia in the scope of the project LA/P/0048/2020. InBIO Barcoding Initiative was funded by the European Union’s Horizon 2020 Research and Innovation Programme under grant agreement No 668981 and the project PORBIOTA—Portuguese E-Infrastructure for Information and Research on Biodiversity (POCI-01-0145- FEDER-022127), supported by Operational Thematic Program for Competitiveness and Internationalization (POCI), under the PORTUGAL 2020 Partnership Agreement, through the European Regional Development Fund (FEDER). Fieldwork benefitted from the project PTDC/ BIA-BIC/2203/2012-FCOMP-01-0124-FEDER-028289 by FEDER Funds through the Operational Programme for Competitiveness Factors–COMPETE and by National Funds, EDP Biodiversity Chair, the project “Promoção dos serviços de ecossistemas no Parque Natural Regional do Vale do Tua: Controlo de Pragas Agrícolas e Florestais por Morcegos” funded by the Agência de Desenvolvimento Regional do Vale doTua and includes research conducted at the Long Term Research Site of Baixo Sabor (LTER_EU_PT_002). The work was partially Funded by Horizon Europe under the Biodiversity, Circular Economy and Environment call (REA.B.3); co-funded by the Swiss State Secretariat for Education, Research and Innovation (SERI) under contract number 22.00173; and by the UK Research and Innovation under the Department for Business, Energy and Industrial Strategy’s Horizon Europe Guarantee Scheme. SF and VM were funded by the FCT through the programme ‘Stimulus of Scientific Employment, Individual Support—3rd Edition’ 2020.03526.CEECIND; 2020.02547.CEECIND). JV and FMSM by PhD grants (SFRH/BD/133159/2017; SFRH/BD/104703/2014) funded by FCT.

## Sampling methods

### Study extent

Continental Portugal (Fig. 3).

### Sampling description

Specimens were collected during field expeditions throughout continental Portugal, from 2005 to 2021. Nearly all districts of continental Portugal are represented in the dataset, with the exception of Braga and Viseu. Setúbal, Beja and Bragança were the districts with the highest number of species collected (Table 2). Specimens were collected by direct search and stored in 96% ethanol. Specimens were kept as tissue samples and stored at the InBIO Barcoding Initiative reference collection (Vairão, Portugal).

DNA extraction and sequencing followed the general pipeline used in the InBIO Barcoding Initiative (Ferreira et al. 2018). Briefly, genomic DNA was extracted from leg tissue using the EasySpin Genomic DNA Tissue Kit (Citomed) following the manufacturer’s protocol. The cytochrome c oxidase I (COI) barcoding fragment (Folmer region) was amplified as two overlapping fragments (LC and BH), using two sets of primers: LCO1490 (Folmer et al. 1994) + Ill_C_R (Shokralla et al. 2015) and Ill_B_F (Shokralla et al. 2015) + HCO2198 (Folmer et al. 1994), respectively. The partial COI mitochondrial gene (Folmer region) was then sequenced in a MiSeq benchtop system. OBITools (https://git.metabarcoding.org/obitools/obitools) was used to process the initial sequences which were then assembled into a single 658 bp fragment using Geneious 9.1.8. (https://www.geneious.com).

### Quality control

All DNA barcodes sequences were compared against the BOLD database and the top hits were inspected to detect possible issues due to contamination or misidentifications due to errors in codification during samples processing.

### Step description

Specimens were collected in 118 different Portuguese localities between 2005 and 2021. Sampling consisted of direct search of specimens in different types of habitats during the day and the night-time. Additionally, some orthopterans were detected by listening to the calling songs. All specimens were morphologically identified using the available literature, DNA barcoded and deposited in the IBI reference collection at CIBIO (Research Center in Biodiversity and Genetic Resources). To sequence the 658 bp COI DNA barcode fragment, one leg was removed from each individual, DNA was extracted and then amplified. All DNA extracts were deposited in the IBI collection. All sequences in the dataset were submitted to BOLD and GenBank databases and, to each sequenced specimen, the morphological identification was compared with the results of the BLAST of the newly-generated DNA barcodes in the BOLD Identification Engine.

## Geographic coverage

### Description

Continental Portugal.

### Coordinates

-9.4651 and -6.5573 Latitude; 42.0416 and 37.019 Longitude.

## Taxonomic coverage

### Description

This dataset is composed of data relating to 420 Orthoptera specimens. All specimens were determined to species level, with four specimens further identified to subspecies level. Overall, 119 species are represented in the dataset. These species belong to all the eleven Orthoptera families occurring in Portugal. The Tettigoniidae and Acrididae families accounts for 36% and 34% of the total collected specimens, respectively, followed by Gryllidae family with 14%. A similar pattern was observed for the proportion of species, Acrididae, Tettigoniidae and Gryllidae are represented by the highest number of recorded species (38%, 34% and 10%, respectively) (Fig. 4).

### Taxa included

**Table:** 

Rank	Scientific Name	
kingdom	Animalia	
phylum	Arthropoda	
class	Insecta	
order	Orthoptera	
family	Acrididae	
family	Gryllidae	
family	Gryllotalpidae	
family	Mogoplistidae	
family	Oecanthidae	
family	Pamphagidae	
family	Pyrgomorphidae	
family	Tetrigidae	
family	Tettigoniidae	
family	Tridactylidae	
family	Trigonidiidae	
subfamily	Acridinae	
subfamily	Bradyporinae	
subfamily	Calliptaminae	
subfamily	Conocephalinae	
subfamily	Cyrtacanthacridinae	
subfamily	Eyprepocnemidinae	
subfamily	Gomphocerinae	
subfamily	Gryllinae	
subfamily	Gryllomorphinae	
subfamily	Gryllotalpinae	
subfamily	Meconematinae	
subfamily	Mogoplistinae	
subfamily	Nemobiinae	
subfamily	Oecanthinae	
subfamily	Oedipodinae	
subfamily	Pamphaginae	
subfamily	Pezotettiginae	
subfamily	Phaneropterinae	
subfamily	Pyrgomorphinae	
subfamily	Tetriginae	
subfamily	Tettigoniinae	
subfamily	Tridactylinae	
subfamily	Trigonidiinae	

## Temporal coverage

**Data range:** 2005-3-13 – 2021-7-09.

### Notes

The sampled material was collected in the period from 13 March 2005 to 9 July 2021.

## Collection data

### Collection name

InBIO Barcoding Initiative

### Specimen preservation method

“Alcohol"

### Curatorial unit

Voucher tube - 1 to 420; DNA extractions - 1 to 420

## Usage licence

### Usage licence

Other

### IP rights notes

Creative Commons Attribution 4.0 International (CC BY 4.0)

## Data resources

### Data package title

The InBIO Barcoding Initiative Database: DNA barcodes of Orthoptera from Portugal

### Resource link

http://dx.doi.org/10.5883/DS-IBIOR01

### Number of data sets

1

### Data set 1.

#### Data set name

Orthoptera

#### Data format

dwc, xml, tsv, fasta

#### Description

The InBIO Barcoding Initiative Database: DNA barcodes of Orthoptera from Portugal dataset can be downloaded from the Public Data Portal of BOLD (https://doi.org/10.5883/DS-IBIOR01) in different formats (data as dwc, xml or tsv and sequences as fasta files). BOLD users can also log-in and access the dataset through the Workbench platform of BOLD. All records are also discoverable within BOLD, using the platform search function. The version of the dataset, at the time of writing the manuscript, is included as Suppl. materials 1, 2, 3 in the form of two text files with specimen data and one fasta file containing all sequences as downloaded from BOLD. Column labels below follow the labels downloaded in the tsv file downloaded from BOLD. Columns with no content in our dataset are left out in the list below.

**Data set 1.** 

Column label	Column description
processid	Unique identifier for the sample.
sampleid	Identifier for the sample being sequenced, i.e. IBI catalogue number at Cibio-InBIO, Porto University. Often identical to the "Field ID" or "Museum ID".
recordID	Identifier for specimen assigned in the field.
catalogNumber	Catalogue number.
fieldnum	Field number.
institution_storing	The full name of the institution that has physical possession of the voucher specimen.
bin_uri	Barcode Index Number system identifier.
phylum_taxID	Phylum taxonomic numeric code.
phylum_name	Phylum name.
class_taxID	Class taxonomic numeric code.
class_name	Class name.
order_taxID	Order taxonomic numeric code.
order_name	Order name.
family_taxID	Family taxonomic numeric code.
family_name	Family name.
subfamily_taxID	Subfamily taxonomic numeric code.
subfamily_name	Subfamily name.
genus_taxID	Genus taxonomic numeric code.
genus_name	Genus name.
species_taxID	Species taxonomic numeric code.
species_name	Species name.
identification_provided_by	Full name of primary individual who assigned the specimen to a taxonomic group.
identification_method	The method used to identify the specimen.
voucher_status	Status of the specimen in an accessioning process (BOLD controlled vocabulary).
tissue_type	A brief description of the type of tissue or material analysed.
collectors	The full or abbreviated names of the individuals or team responsible for collecting the sample in the field.
lifestage	The age class or life stage of the specimen at the time of sampling.
sex	The sex of the specimen.
lat	The geographical latitude (in decimal degrees) of the geographic centre of a location.
lon	The geographical longitude (in decimal degrees) of the geographic centre of a location.
elev	Elevation of sampling site (in metres above sea level).
country	The full, unabbreviated name of the country where the organism was collected.
province_state	The full, unabbreviated name of the province ("Distrito" in Portugal) where the organism was collected.
region	The full, unabbreviated name of the municipality ("Concelho" in Portugal) where the organism was collected.
exactsite	Additional name/text description regarding the exact location of the collection site relative to a geographic relevant landmark.
subspecies_taxID	Subspecies taxonomic numeric code.
subspecies_name	Subspecies name.

## Supplementary Material

2A9F5A6F-215C-5DE2-8F11-FB7803674300 10.3897/BDJ.12.e118010.suppl1 Supplementary material 1 DS-IBIOR01 InBIO Barcoding Initiative - Orthoptera 01 - Specimen details Data type Record information - specimen data Brief description The file includes information about all records in BOLD for the DS-IBIOR01 InBIO Barcoding Initiative - Orthoptera 01 library. It contains collection and identification data. File: oo_959988.txt https://binary.pensoft.net/file/959988 Sílvia Pina, Joana Paupério, Francisco Barros, Cátia Chaves, Filipa M.S. Martins, Joana Pinto, Joana Veríssimo, Vanessa Mata, Pedro Beja, Sónia Ferreira

5E5ABC10-1510-542C-BC45-F5007AB97837 10.3897/BDJ.12.e118010.suppl2 Supplementary material 2 IBI – Orthoptera 01 library - Specimen details - Darwin Core Standard Data type Specimen data records in the Darwin Core Standard format. Brief description The file includes information about all records in BOLD for the DS-IBIOR01 InBIO Barcoding Initiative - Orthoptera 01. It contains collection and identification data. File: oo_959989.txt https://binary.pensoft.net/file/959989 Sílvia Pina, Joana Paupério, Francisco Barros, Cátia Chaves, Filipa M.S. Martins, Joana Pinto, Joana Veríssimo, Vanessa Mata, Pedro Beja, Sónia Ferreira

0AE860CE-D7A8-504B-9FE1-2BE1B7E608C5 10.3897/BDJ.12.e118010.suppl3 Supplementary material 3 IBI - Orthoptera 01 library - DNA sequences Data type Specimen genomic data, DNA sequences. Brief description COI sequences in fasta format. Each sequence is identified by the BOLD ProcessID, species name, genetic marker name and GenBank accession number, all separated by a vertical bar. The data are as downloaded from BOLD. File: oo_959303.fas https://binary.pensoft.net/file/959303 Sílvia Pina, Joana Paupério, Francisco Barros, Cátia Chaves, Filipa M.S. Martins, Joana Pinto, Joana Veríssimo, Vanessa Mata, Pedro Beja, Sónia Ferreira

## Figures and Tables

**Figure 1a.**
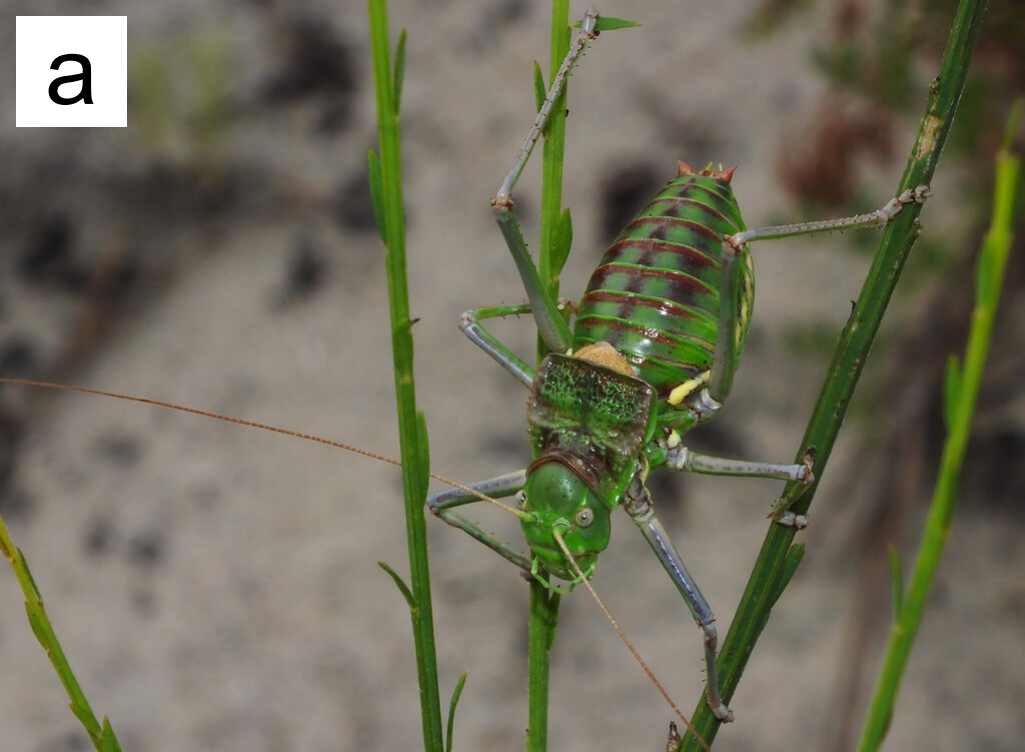
*Neocallicraniabarrosi* Barat, 2013. Tettigoniidae. Photo by Francisco Barros. BIN URI BOLD:AEO7827;

**Figure 1b.**
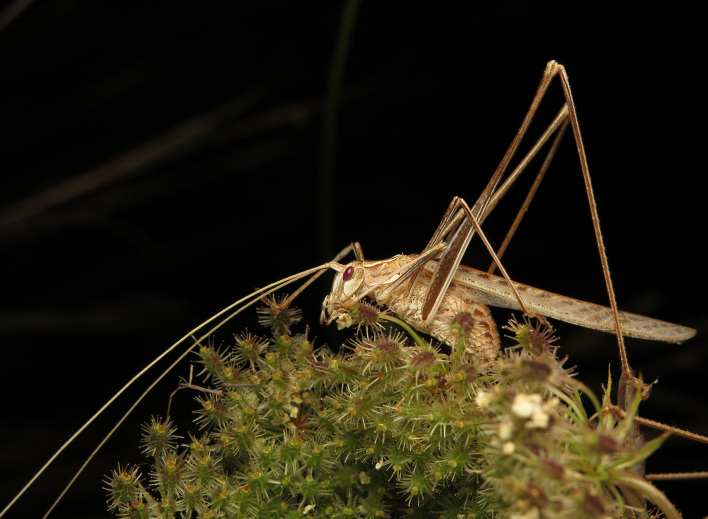
*Tylopsislilifolia* (Fabricius, 1793). Tettigoniidae. Photo by Sónia Ferreira. BIN URI BOLD:AER2541;

**Figure 1c.**
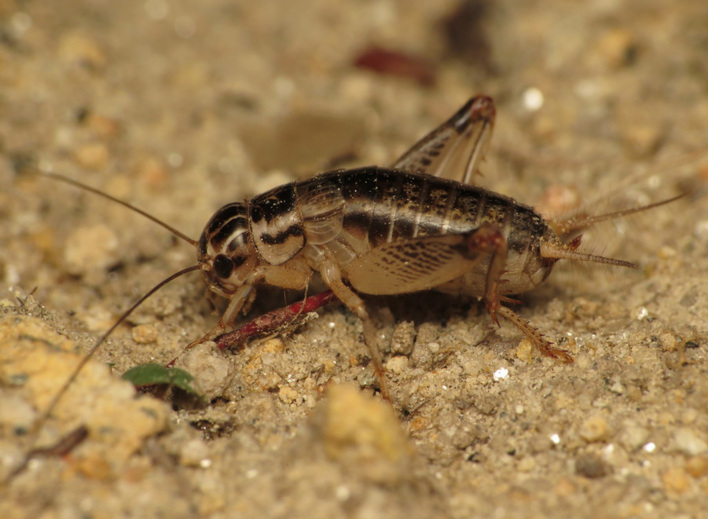
*Eugryllodesescalerae* (Bolívar, 1894). Gryllidae. Photo by Sónia Ferreira. BIN URI BOLD:AEP5287;

**Figure 1d.**
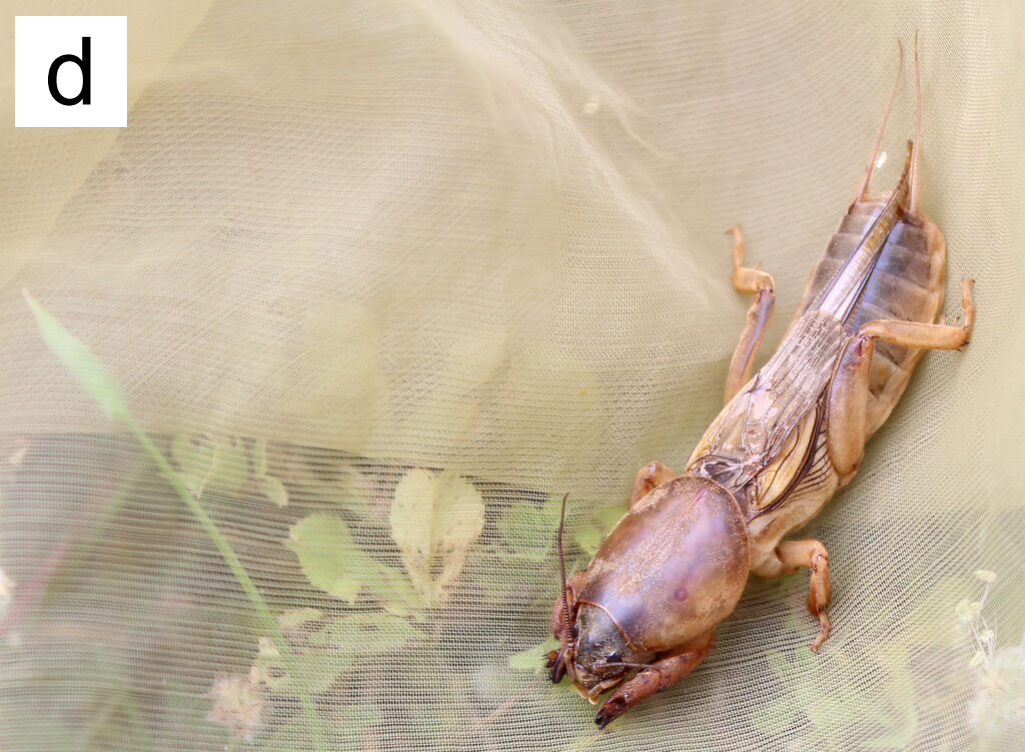
*Gryllotalpavineae* Bennet-Clark, 1970. Gryllotalpidae. Photo by Sílvia Pina. BIN URI BOLD:AEP5099; BOLD:AEP5100;

**Figure 1e.**
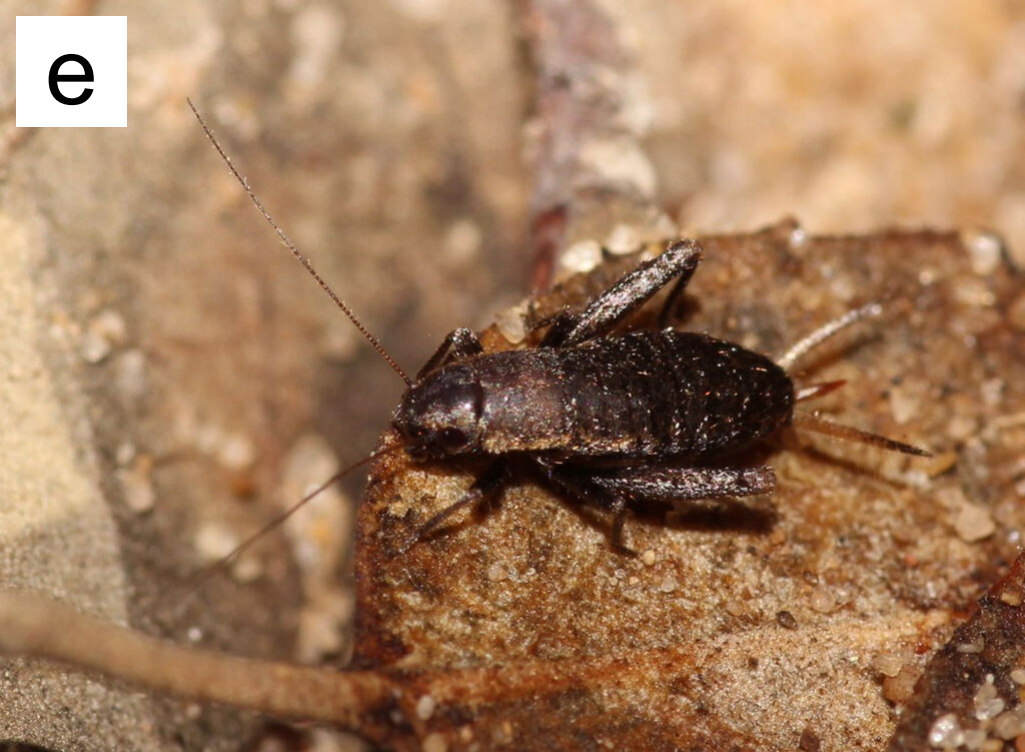
*Paramogoplistesortini* Llucià Pomares, 2015. Mogoplistidae. Photo by Sílvia Pina. BIN URI BOLD:AEO7942;

**Figure 1f.**
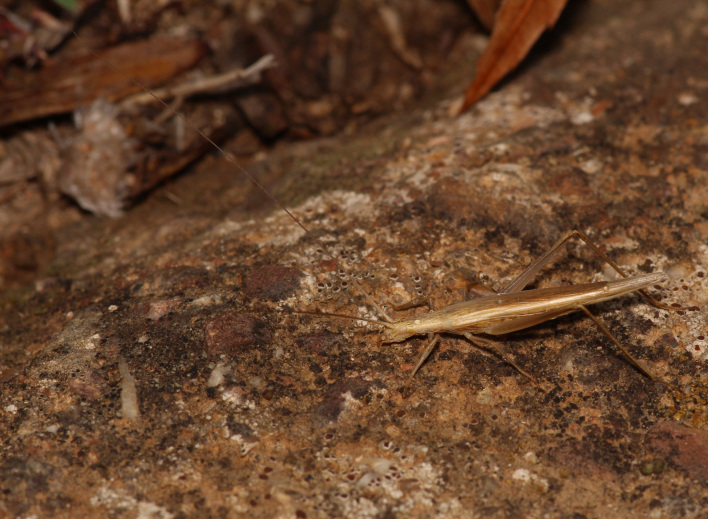
*Oecanthusdulcisonans* Gorochov, 1993. Oecanthidae. Photo by Sílvia Pina. BIN URI BOLD:ACG7286.

**Figure 2a.**
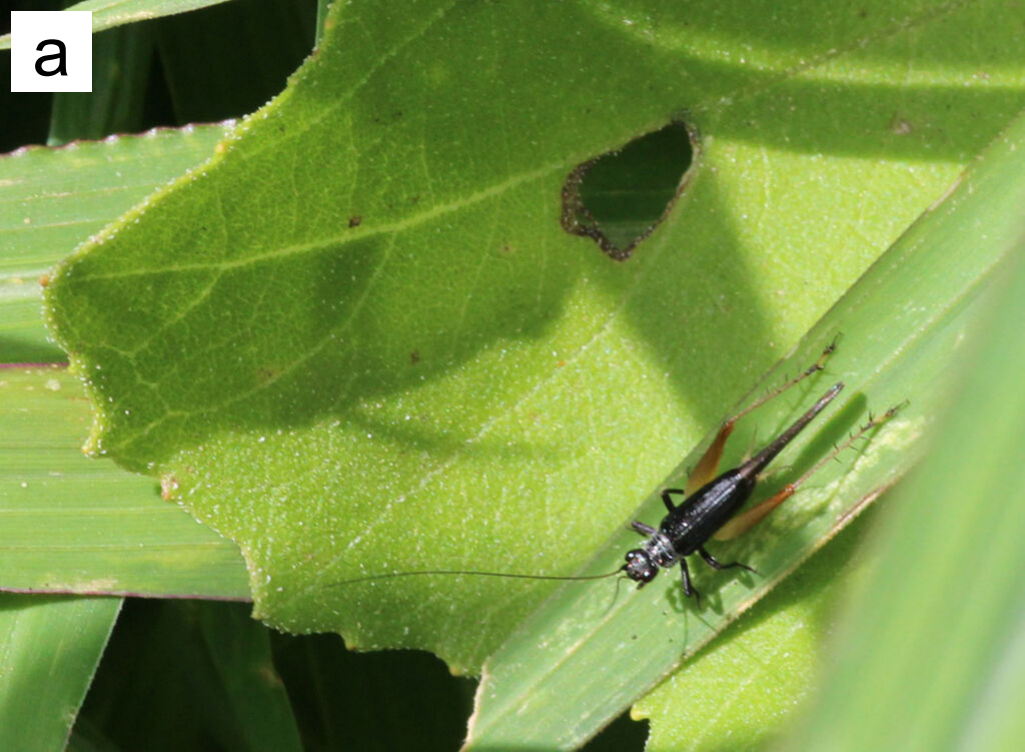
*Trigonidiumcicindeloides* Rambur, 1838. Trigonidiidae. Photo by Sílvia Pina. BIN URI BOLD:ACY3774;

**Figure 2b.**
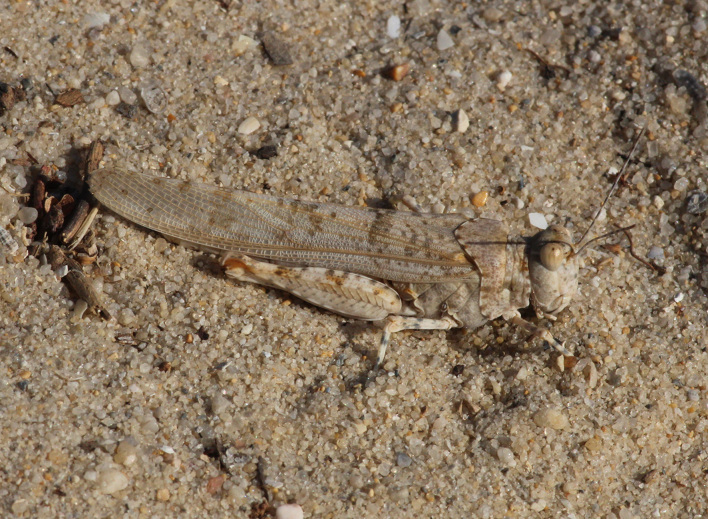
*Sphingonotuslusitanicus* Ebner, 1941. Acrididae. Photo by Sílvia Pina. BIN URI BOLD:AEI2886;

**Figure 2c.**
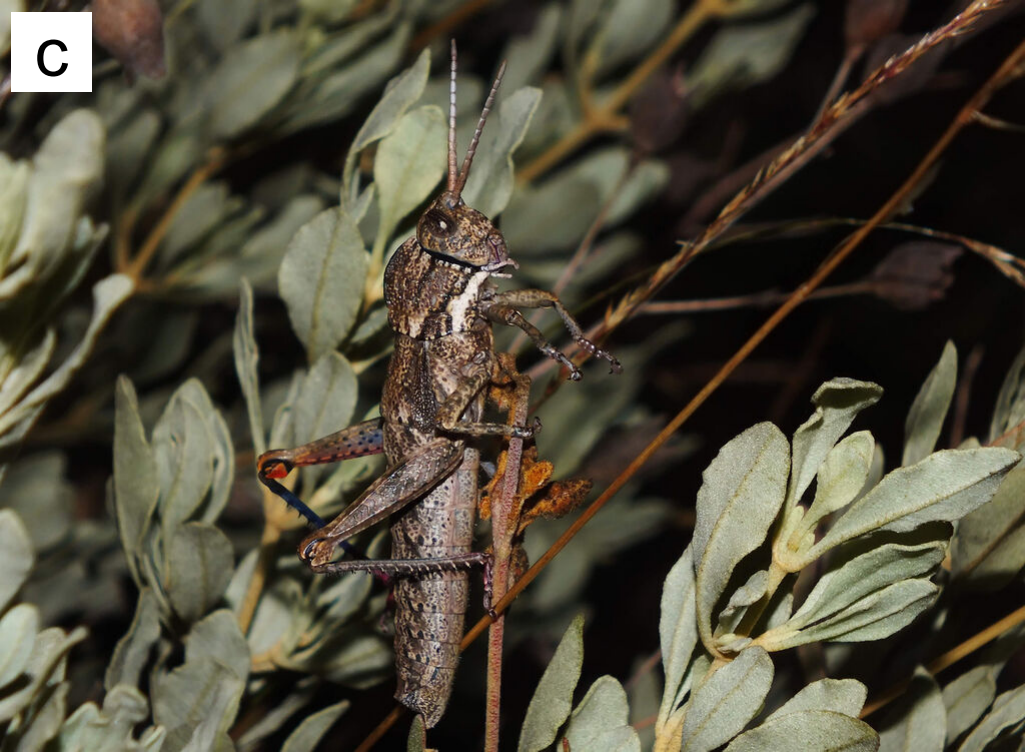
*Acinipeignatii* Llorente del Moral & Presa, 1983. Pamphagidae. Photo by Francisco Barros. BIN URI BOLD:AER2325;

**Figure 2d.**
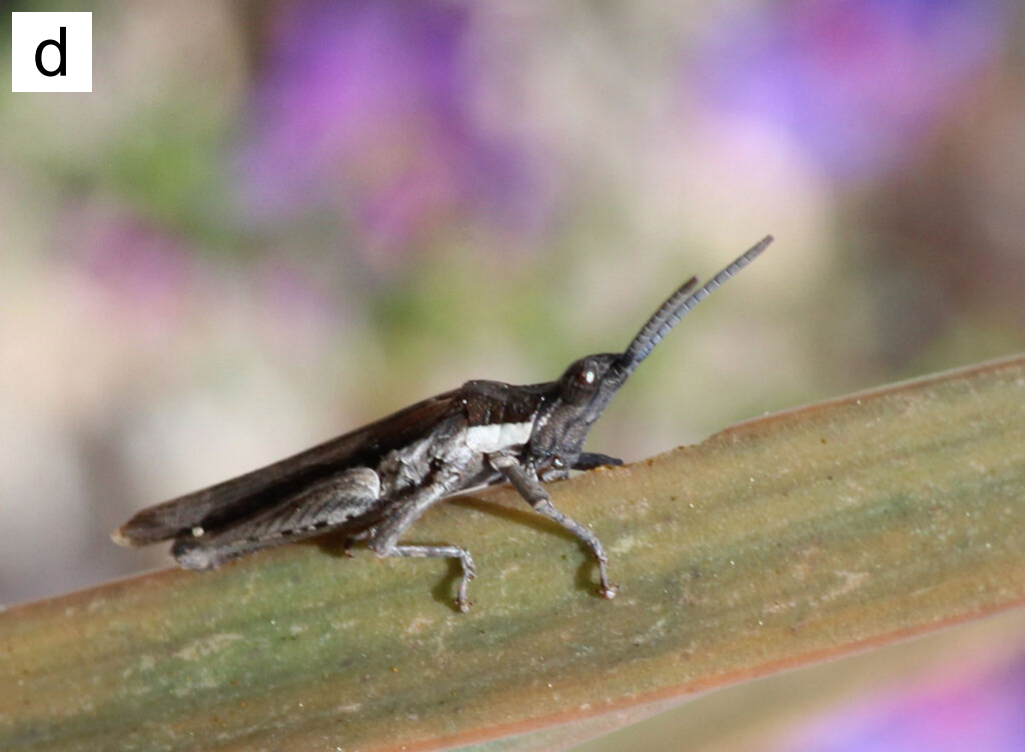
*Pyrgomorphaconica* (Olivier, 1791). Pyrgomorphidae. Photo by Sílvia Pina. BIN URI BOLD:AEO6938;

**Figure 2e.**
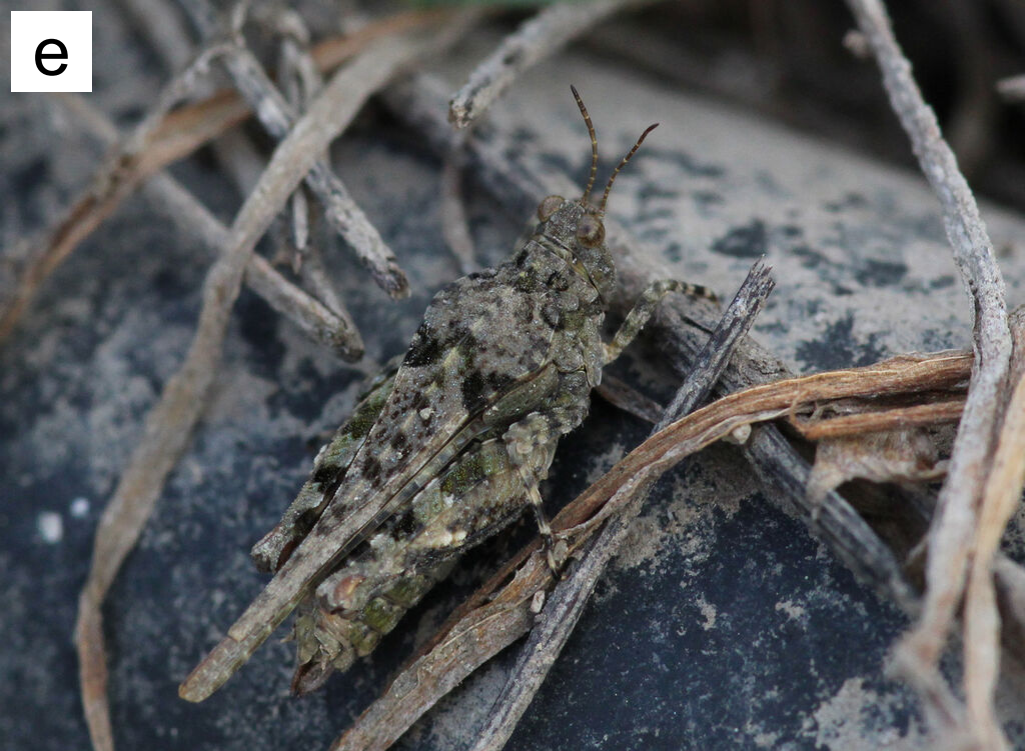
*Paratettixmeridionalis* (Rambur, 1838). Tetrigidae. Photo by Sílvia Pina. BIN URI BOLD:BOLD:AAP5007;

**Figure 2f.**
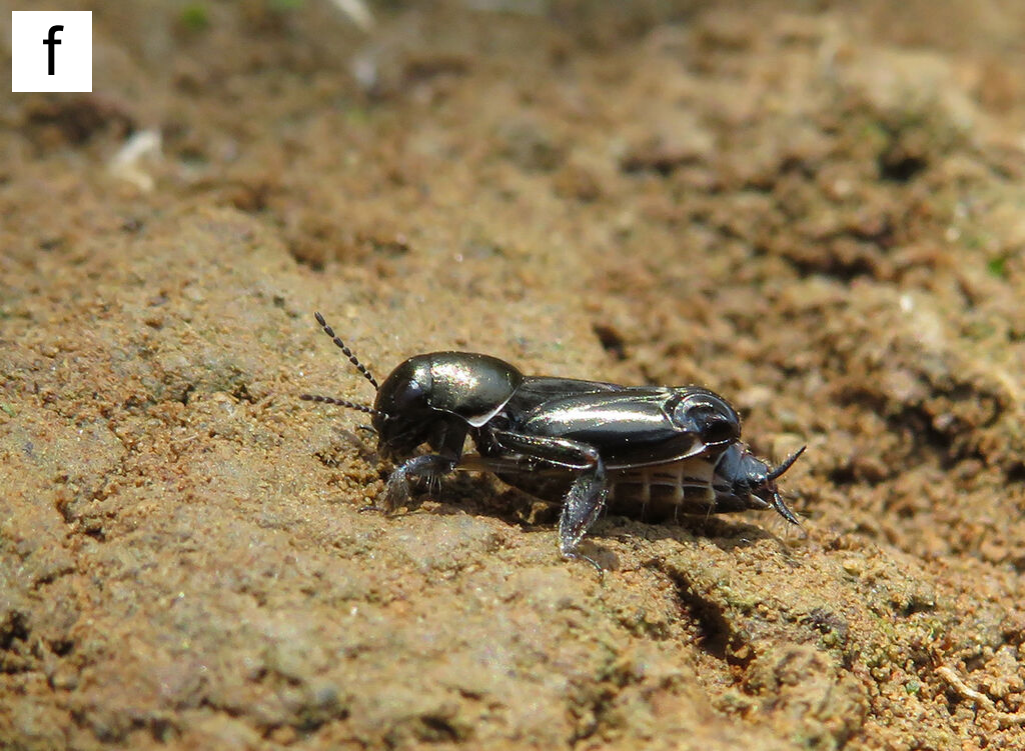
*Xyaiberica* Günther, 1990. Tridactylidae. Photo by Sónia Ferreira. BIN URI BOLD:AER2238.

**Figure 4a.**
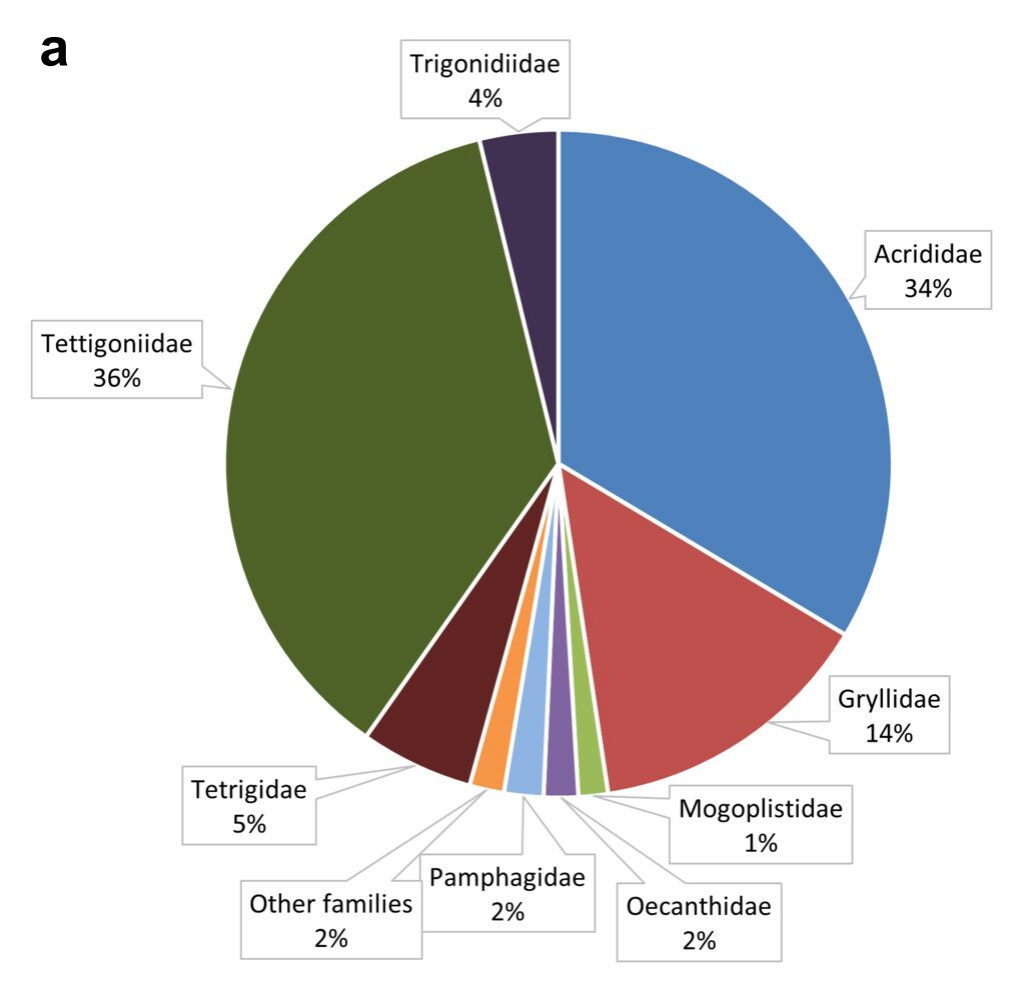
Distribution of specimens, in percentage, per Orthoptera family present in the dataset.

**Figure 4b.**
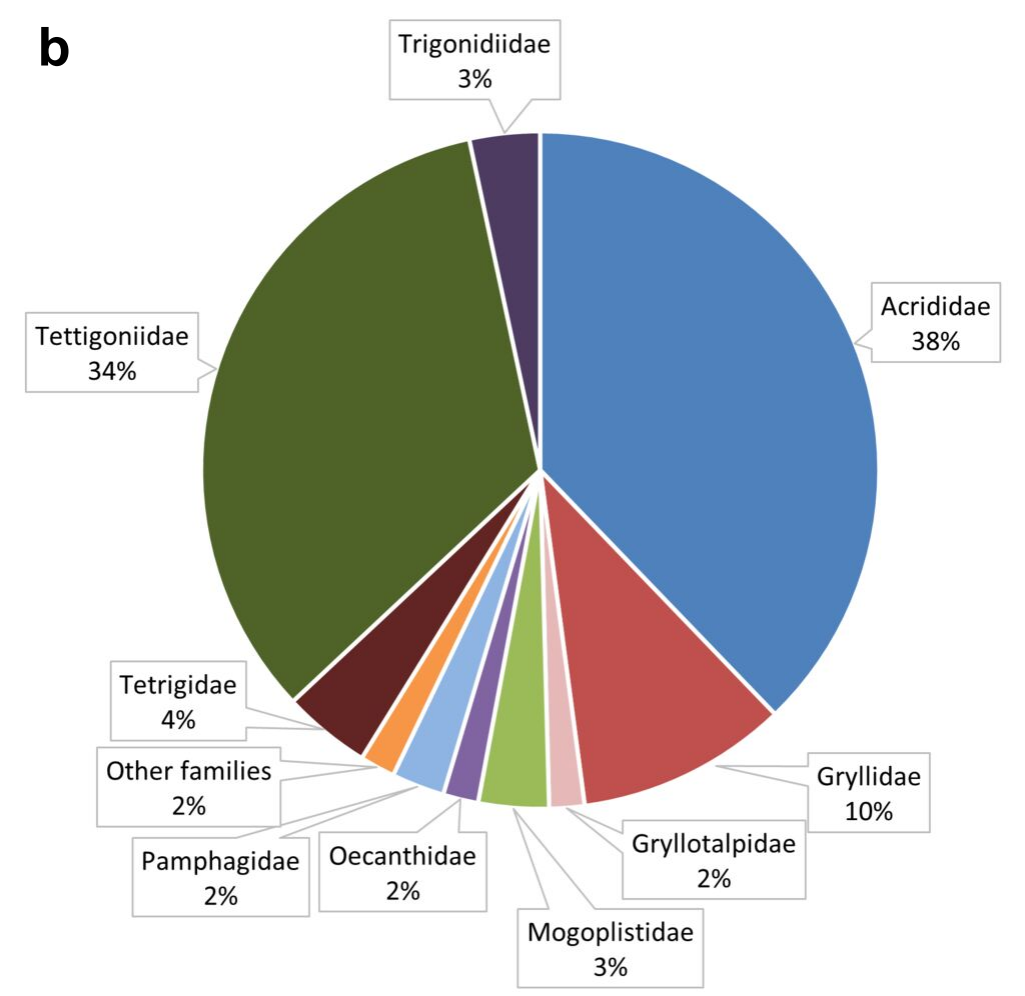
Distribution of species, in percentage, per Orthoptera family present in the dataset.

**Table 1.** List of taxa that were collected and DNA barcoded within this project. In column Taxa: ^1^ - indicates Iberian endemic species; ^2^ - indicates continental Portugal endemic species; ^#^ - indicates taxa without a public DNA barcode prior to this study; " - indicates unique BINs.

**Family**	**Taxa**	**IBI code**	**BOLD code**	**BOLD BIN**	**GenBank**
Acrididae	*Acrotylusinsubricus* (Scopoli, 1786)	INV02543	IBIOR108-17	BOLD:AAP4452	OR974638
Acrididae	*Acrotylusinsubricus* (Scopoli, 1786)	INV02544	IBIOR109-17	BOLD:AAP4452	OR974794
Acrididae	*Acrotylusinsubricus* (Scopoli, 1786)	INV05545	IBIOR196-22	BOLD:AAP4452	OR974607
Acrididae	*Acrotylusinsubricus* (Scopoli, 1786)	INV06642	IBIOR436-22	BOLD:AAP4452	OR974455
Acrididae	*Acrotyluspatruelis* (Herrich-Schäffer, 1838)	INV00063	IBIOR002-17	BOLD:AAJ6293	OR974610
Acrididae	*Acrotyluspatruelis* (Herrich-Schäffer, 1838)	INV03740	IBIOR326-22	BOLD:AAJ6293	OR974601
Acrididae	*Acrotyluspatruelis* (Herrich-Schäffer, 1838)	INV05270	IBIOR392-22	BOLD:AAJ6293	OR974494
Acrididae	*Acrotyluspatruelis* (Herrich-Schäffer, 1838)	INV10594	IBIOR535-22	BOLD:AAJ6293	OR974432
Acrididae	*Aiolopuspuissanti* Defaut, 2005	INV00065	IBIOR004-17	BOLD:AAZ1689	OR974633
Acrididae	*Aiolopuspuissanti* Defaut, 2005	INV02502	IBIOR067-17	BOLD:AAZ1689	OR974542
Acrididae	*Aiolopusstrepens* (Latreille, 1804)	INV00066	IBIOR005-17	BOLD:AAP4386	OR974796
Acrididae	*Aiolopusstrepens* (Latreille, 1804)	INV02503	IBIOR068-17	BOLD:AAP4386	OR974414
Acrididae	*Aiolopusstrepens* (Latreille, 1804)	INV02539	IBIOR104-17	BOLD:AAP4386	OR974707
Acrididae	*Aiolopusstrepens* (Latreille, 1804)	INV02540	IBIOR105-17	BOLD:AAP4386	OR974749
Acrididae	*Aiolopusstrepens* (Latreille, 1804)	INV04053	IBIOR351-22	BOLD:AAP4386	OR974447
Acrididae	*Aiolopusstrepens* (Latreille, 1804)	INV06052	IBIOR406-22	BOLD:AAP4386	OR974623
Acrididae	*Anacridiumaegyptium* (Linnaeus, 1764)	INV02504	IBIOR069-17	BOLD:AAO1248	OR974491
Acrididae	*Anacridiumaegyptium* (Linnaeus, 1764)	INV02505	IBIOR070-17	BOLD:AAO1248	OR974554
Acrididae	*Anacridiumaegyptium* (Linnaeus, 1764)	INV04117	IBIOR166-19	BOLD:AAO1248	OR974781
Acrididae	*Arcypteratornosi* Bolívar, 18841	INV02598	IBIOR188-22	BOLD:AER6128	OR974776
Acrididae	*Arcypteratornosi* Bolívar, 18841	INV02599	IBIOR189-22	BOLD:AER6128	OR974669
Acrididae	*Calephoruscompressicornis* (Latreille, 1804)	INV02562	IBIOR120-17	BOLD:AEA6681	OR974480
Acrididae	*Calephoruscompressicornis* (Latreille, 1804)	INV02563	IBIOR121-17	BOLD:AEA6681	OR974777
Acrididae	*Calliptamusbarbarus* (Costa, 1836)	INV00067	IBIOR006-17	BOLD:ACQ0538	OR974431
Acrididae	*Calliptamusbarbarus* (Costa, 1836)	INV00068	IBIOR007-17	BOLD:ACQ0538	OR974606
Acrididae	*Calliptamusbarbarus* (Costa, 1836)	INV02550	IBIOR113-17	BOLD:ACQ0538	OR974538
Acrididae	*Calliptamusbarbarus* (Costa, 1836)	INV03646	IBIOR314-22	BOLD:ACQ0538	OR974580
Acrididae	*Calliptamusbarbarus* (Costa, 1836)	INV04028	IBIOR346-22	BOLD:ACQ0538	OR974686
Acrididae	*Calliptamuswattenwylianus* Pantel, 1896	INV00069	IBIOR008-17	BOLD:AEZ7204	OR974823
Acrididae	*Calliptamuswattenwylianus* Pantel, 1896	INV00070	IBIOR009-17	BOLD:AEZ7204	OR974681
Acrididae	*Calliptamuswattenwylianus* Pantel, 1896	INV11426	IBIOR550-22	BOLD:AEZ7204	OR974745
Acrididae	*Chorthippusapicalis* (Herrich-Schäffer, 1840)	INV00071	IBIOR010-17	BOLD:AED8559	OR974762
Acrididae	*Chorthippusapicalis* (Herrich-Schäffer, 1840)	INV00072	IBIOR011-17	BOLD:AED8559	OR974600
Acrididae	*Chorthippusapicalis* (Herrich-Schäffer, 1840)	INV02506	IBIOR071-17	BOLD:AED8559	OR974536
Acrididae	*Chorthippusapicalis* (Herrich-Schäffer, 1840)	INV02507	IBIOR072-17	BOLD:AED8559	OR974696
Acrididae	*Chorthippusapicalis* (Herrich-Schäffer, 1840)	INV02532	IBIOR097-17	BOLD:AED8559	OR974673
Acrididae	*Chorthippusapicalis* (Herrich-Schäffer, 1840)	INV02533	IBIOR098-17	BOLD:AED8559	OR974657
Acrididae	*Chorthippusbinotatus* (Charpentier, 1825)	INV03941	IBIOR331-22	BOLD:AAC5779	OR974519
Acrididae	*Chorthippusbinotatus* (Charpentier, 1825)	INV05546	IBIOR197-22	BOLD:AAC5779	OR974508
Acrididae	*Chorthippusbinotatus* (Charpentier, 1825)	INV05547	IBIOR198-22	BOLD:AAC5779	OR974733
Acrididae	*Chorthippusjacobsi* Harz, 19751	INV02535	IBIOR100-17	BOLD:AAC5779	OR974828
Acrididae	*Chorthippusjacobsi* Harz, 19751	INV02536	IBIOR101-17	BOLD:AAC5779	OR974425
Acrididae	*Chorthippusjacobsi* Harz, 19751	INV02537	IBIOR102-17	BOLD:AAC5779	OR974577
Acrididae	*Chorthippusjucundus* (Fischer, 1853)	INV03734	IBIOR323-22	BOLD:AER0035"	OR974582
Acrididae	*Chorthippusvagans* (Eversmann, 1848)	INV00073	IBIOR012-17	BOLD:AAL3938	OR974714
Acrididae	*Chorthippusvagans* (Eversmann, 1848)	INV00074	IBIOR013-17	BOLD:AAL3938	OR974540
Acrididae	*Chorthippusvagans* (Eversmann, 1848)	INV02548	IBIOR111-17	BOLD:AAL3938	OR974820
Acrididae	*Chorthippusvagans* (Eversmann, 1848)	INV02704	IBIOR571-22	BOLD:AAL3938	OR974618
Acrididae	*Chorthippusyersini* Harz, 19751	INV05556	IBIOR207-22	BOLD:AAC5779	OR974682
Acrididae	*Chorthippusyersini* Harz, 19751	INV05557	IBIOR208-22	BOLD:AAC5779	OR974805
Acrididae	*Dociostaurusgenei* (Ocskay, 1832)	INV00075	IBIOR014-17	BOLD:ADU3247	OR974636
Acrididae	*Dociostaurusgenei* (Ocskay, 1832)	INV00076	IBIOR015-17	BOLD:ADU3247	OR974806
Acrididae	*Dociostaurushispanicus* Bolívar, 18981	INV00079	IBIOR018-17	BOLD:ADV6985	OR974787
Acrididae	*Dociostaurusjagoi* Soltani, 1978	INV00077	IBIOR016-17	BOLD:ADT8663	OR974505
Acrididae	*Dociostaurusjagoi* Soltani, 1978	INV00078	IBIOR017-17	BOLD:ADT8663	OR974567
Acrididae	*Dociostaurusjagoi* Soltani, 1978	INV02549	IBIOR112-17	BOLD:ADT8663	OR974476
Acrididae	*Dociostaurusjagoi* Soltani, 1978	INV03536	IBIOR308-22	BOLD:ADT8663	OR974516
Acrididae	*Dociostaurusjagoi* Soltani, 1978	INV03540	IBIOR191-22	BOLD:ADT8663	OR974736
Acrididae	*Dociostaurusjagoi* Soltani, 1978	INV03547	IBIOR309-22	BOLD:ADT8663	OR974585
Acrididae	*Dociostaurusjagoi* Soltani, 1978	INV03556	IBIOR310-22	BOLD:ADT8663	OR974495
Acrididae	*Dociostaurusjagoi* Soltani, 1978	INV03628	IBIOR313-22	BOLD:ADT8663	OR974808
Acrididae	*Dociostaurusjagoi* Soltani, 1978	INV06726	IBIOR438-22	BOLD:ADT8663	OR974514
Acrididae	*Dociostaurusjagoi* Soltani, 1978	INV07282	IBIOR464-22	BOLD:ADT8663	OR974807
Acrididae	*Dociostaurusjagoi* Soltani, 1978	INV07542	IBIOR473-22	BOLD:ADT8663	OR974544
Acrididae	*Dociostaurusjagoi* Soltani, 1978	INV07619	IBIOR480-22	BOLD:ADT8663	OR974532
Acrididae	*Dociostaurusjagoi* Soltani, 1978	INV11390	IBIOR546-22	BOLD:ADT8663	OR974482
Acrididae	*Dociostaurusjagoi* Soltani, 1978	INV11391	IBIOR547-22	BOLD:ADT8663	OR974635
Acrididae	*Dociostaurusjagoi* Soltani, 1978	INV11393	IBIOR549-22	BOLD:ADT8663	OR974646
Acrididae	*Dociostaurusmaroccanus* (Thunberg, 1815)	INV00080	IBIOR019-17	BOLD:AAX8673	OR974534
Acrididae	*Dociostaurusmaroccanus* (Thunberg, 1815)	INV00081	IBIOR020-17	BOLD:AAX8673	OR974574
Acrididae	*Euchorthippuselegantulus* Zeuner, 1940#	INV00083	IBIOR021-17	BOLD:ADJ5172	OR974465
Acrididae	*Euchorthippuselegantulus* Zeuner, 1940#	INV00084	IBIOR022-17	BOLD:ADJ5172	OR974592
Acrididae	*Euchorthippuselegantulus* Zeuner, 1940#	INV00085	IBIOR023-17	BOLD:ADJ5172	OR974659
Acrididae	*Euchorthippuselegantulus* Zeuner, 1940#	INV00086	IBIOR024-17	BOLD:ADJ5172	OR974625
Acrididae	*Euchorthippuselegantulus* Zeuner, 1940#	INV00087	IBIOR025-17	BOLD:ADJ5172	OR974559
Acrididae	*Euchorthippuselegantulus* Zeuner, 1940#	INV00088	IBIOR026-17	BOLD:ADJ5172	OR974666
Acrididae	*Euchorthippuselegantulus* Zeuner, 1940#	INV02579	IBIOR135-17	BOLD:ADJ5172	OR974674
Acrididae	*Euchorthippuselegantulus* Zeuner, 1940#	INV02580	IBIOR136-17	BOLD:ADJ5172	OR974803
Acrididae	*Euchorthippuselegantulus* Zeuner, 1940#	INV03557	IBIOR311-22	BOLD:ADJ5172	OR974428
Acrididae	*Eyprepocnemisplorans* (Charpentier, 1825)	INV05585	IBIOR236-22	BOLD:AAV4939	OR974729
Acrididae	*Eyprepocnemisplorans* (Charpentier, 1825)	INV05586	IBIOR237-22	BOLD:AAV4939	OR974676
Acrididae	*Locustamigratoria* (Linnaeus, 1758)	INV02510	IBIOR075-17	BOLD:AAD9526	OR974584
Acrididae	*Locustamigratoria* (Linnaeus, 1758)	INV02511	IBIOR076-17	BOLD:AAD9526	OR974821
Acrididae	*Locustamigratoria* (Linnaeus, 1758)	INV05513	IBIOR141-19	BOLD:AAD9526	OR974698
Acrididae	*Mioscirtuswagneri* (Eversmann, 1859)	INV05564	IBIOR215-22	BOLD:ABV1949	OR974436
Acrididae	*Mioscirtuswagneri* (Eversmann, 1859)	INV05565	IBIOR216-22	BOLD:ABV1949	OR974457
Acrididae	*Morphacrisfasciata* (Thunberg, 1815)	INV02560	IBIOR118-17	BOLD:ACR8091	OR974668
Acrididae	*Morphacrisfasciata* (Thunberg, 1815)	INV02569	IBIOR125-17	BOLD:ACR8091	OR974488
Acrididae	*Oedaleusdecorus* (Germar, 1825)	INV00091	IBIOR027-17	BOLD:AAE5868	OR974713
Acrididae	*Oedaleusdecorus* (Germar, 1825)	INV00092	IBIOR142-19	BOLD:AAE5868	OR974497
Acrididae	*Oedipodacaerulescens* (Linnaeus, 1758)	INV00093	IBIOR028-17	BOLD:AAC9181	OR974732
Acrididae	*Oedipodacaerulescens* (Linnaeus, 1758)	INV00094	IBIOR029-17	BOLD:AAC9181	OR974612
Acrididae	*Oedipodacaerulescens* (Linnaeus, 1758)	INV02551	IBIOR114-17	BOLD:AAC9181	OR974535
Acrididae	*Oedipodacaerulescens* (Linnaeus, 1758)	INV04033	IBIOR161-19	BOLD:AAC9181	OR974793
Acrididae	*Oedipodacharpentieri* Fieber, 1853	INV00095	IBIOR030-17	BOLD:AAC9180	OR974452
Acrididae	*Oedipodacharpentieri* Fieber, 1853	INV00096	IBIOR031-17	BOLD:AAC9180	OR974742
Acrididae	*Oedipodacharpentieri* Fieber, 1853	INV02512	IBIOR077-17	BOLD:AAC9180	OR974548
Acrididae	*Oedipodacharpentieri* Fieber, 1853	INV02513	IBIOR078-17	BOLD:AAC9180	OR974571
Acrididae	*Oedipodacoerulea* Saussure, 1884#	INV07299	IBIOR465-22	BOLD:AEO7756	OR974419
Acrididae	*Omocestuspanteli* (Bolívar, 1887)^1^	INV00097	IBIOR032-17	BOLD:AAX0669	OR974413
Acrididae	*Omocestuspanteli* (Bolívar, 1887)^1^	INV00098	IBIOR033-17	BOLD:AAX0669	OR974740
Acrididae	*Omocestuspanteli* (Bolívar, 1887)^1^	INV00099	IBIOR034-17	BOLD:AAX0669	OR974780
Acrididae	*Omocestuspanteli* (Bolívar, 1887)^1^	INV00100	IBIOR035-17	BOLD:AAX0669	OR974546
Acrididae	*Omocestuspanteli* (Bolívar, 1887)^1^	INV00101	IBIOR036-17	BOLD:AAX0669	OR974726
Acrididae	*Omocestuspanteli* (Bolívar, 1887)^1^	INV02531	IBIOR096-17	BOLD:AAX0669	OR974790
Acrididae	*Omocestuspanteli* (Bolívar, 1887)^1^	INV10730	IBIOR265-22	BOLD:AAX0669	OR974565
Acrididae	*Omocestusraymondi* (Yersin, 1863)	INV02588	IBIOR178-22	BOLD:AEO7761	OR974430
Acrididae	*Omocestusraymondi* (Yersin, 1863)	INV02589	IBIOR179-22	BOLD:AEO7761	OR974511
Acrididae	*Omocestusrufipes* (Zetterstedt, 1821)	INV02575	IBIOR131-17	BOLD:AAE1108	OR974755
Acrididae	*Omocestusrufipes* (Zetterstedt, 1821)	INV02576	IBIOR132-17	BOLD:AAE1108	OR974773
Acrididae	*Omocestusviridulus* (Linnaeus, 1758)	INV05555	IBIOR206-22	BOLD:AAE1108	OR974599
Acrididae	*Paracinematricolor* (Thunberg, 1815)	INV06628	IBIOR432-22	BOLD:ACU5079	OR974730
Acrididae	*Pezotettixgiornae* (Rossi, 1794)	INV00102	IBIOR037-17	BOLD:AEO7437"	OR974583
Acrididae	*Pezotettixgiornae* (Rossi, 1794)	INV00103	IBIOR038-17	BOLD:AEO7437"	OR974543
Acrididae	*Pezotettixgiornae* (Rossi, 1794)	INV02538	IBIOR103-17	BOLD:AEO7437"	OR974709
Acrididae	*Pezotettixgiornae* (Rossi, 1794)	INV03278	IBIOR306-22	BOLD:AEO7436	OR974802
Acrididae	*Pseudochorthippusparallelus* (Zetterstedt, 1821)	INV03712	IBIOR319-22	BOLD:AAC3399	OR974527
Acrididae	*Pseudochorthippusparallelus* (Zetterstedt, 1821)	INV05554	IBIOR205-22	BOLD:AAC3399	OR974441
Acrididae	*Sphingonotusazurescens* (Rambur, 1838)^1^	INV02556	IBIOR116-17	BOLD:AAJ3344	OR974768
Acrididae	*Sphingonotusazurescens* (Rambur, 1838)^1^	INV02557	IBIOR172-22	BOLD:AAJ3344	OR974647
Acrididae	*Sphingonotusazurescens* (Rambur, 1838)^1^	INV02594	IBIOR184-22	BOLD:AAJ3344	OR974570
Acrididae	*Sphingonotusazurescens* (Rambur, 1838)^1^	INV06744	IBIOR439-22	BOLD:AAJ3344	OR974738
Acrididae	*Sphingonotusimitans* Brunner von Wattenwyl, 1882^1,^#	INV02555	IBIOR171-22	BOLD:AER3084"	OR974661
Acrididae	*Sphingonotusimitans* Brunner von Wattenwyl, 1882^1,^#	INV08093	IBIOR244-22	BOLD:AER3084"	OR974539
Acrididae	*Sphingonotuslluciapomaresi* (Defaut, 2005)^1^	INV00105	IBIOR040-17	BOLD:AEI2886	OR974734
Acrididae	*Sphingonotuslluciapomaresi* (Defaut, 2005)^1^	INV00106	IBIOR041-17	BOLD:AEI2886	OR974489
Acrididae	*Sphingonotuslluciapomaresi* (Defaut, 2005)^1^	INV00107	IBIOR042-17	BOLD:AEI2886	OR974721
Acrididae	*Sphingonotuslluciapomaresi* (Defaut, 2005)^1^	INV03709	IBIOR316-22	BOLD:AEI2886	OR974433
Acrididae	*Sphingonotuslluciapomaresi* (Defaut, 2005)^1^	INV03711	IBIOR318-22	BOLD:AEI2886	OR974624
Acrididae	*Sphingonotuslluciapomaresi* (Defaut, 2005)^1^	INV11392	IBIOR548-22	BOLD:AEI2886	OR974569
Acrididae	*Sphingonotuslusitanicus* Ebner, 1941^1,^#	INV08094	IBIOR245-22	BOLD:AEI2886	OR974699
Acrididae	*Sphingonotusnodulosus* Llucià Pomares, 2013^1,^#	INV02553	IBIOR147-19	BOLD:AAJ3344	OR974506
Acrididae	*Sphingonotusnodulosus* Llucià Pomares, 2013^1^,#	INV02596	IBIOR186-22	BOLD:AAJ3344	OR974703
Acrididae	*Sphingonotusrubescens* (Walker, 1870)	INV02591	IBIOR181-22	BOLD:AFB6849	OR974611
Acrididae	*Stenobothrusgrammicus* Cazurro y Ruiz, 1888	INV08096	IBIOR247-22	BOLD:AER4390	OR974501
Acrididae	*Stenobothrusgrammicus* Cazurro y Ruiz, 1888	INV08097	IBIOR248-22	BOLD:AER4390	OR974737
Acrididae	*Stenobothruslineatus* (Panzer, 1796)	INV05566	IBIOR217-22	BOLD:AAC6368	OR974812
Acrididae	*Stenobothrusstigmaticus* (Rambur, 1838)	INV05552	IBIOR203-22	BOLD:AAD9833	OR974708
Acrididae	*Stenobothrusstigmaticus* (Rambur, 1838)	INV05558	IBIOR209-22	BOLD:AAD9833	OR974772
Acrididae	*Truxalisnasuta* (Linnaeus, 1758)#	INV02522	IBIOR087-17	BOLD:AAP6089	OR974692
Acrididae	*Truxalisnasuta* (Linnaeus, 1758)#	INV02523	IBIOR088-17	BOLD:AAP6089	OR974525
Gryllidae	*Achetadomesticus* (Linnaeus, 1758)	INV06916	IBIOR443-22	BOLD:AAJ7534	OR974760
Gryllidae	*Achetadomesticus* (Linnaeus, 1758)	INV06947	IBIOR452-22	BOLD:AAJ7534	OR974811
Gryllidae	*Achetahispanicus* Rambur, 1838#	INV06943	IBIOR448-22	BOLD:AAP6118	OR974604
Gryllidae	*Achetahispanicus* Rambur, 1838#	INV11450	IBIOR553-22	BOLD:AAP6118	OR974517
Gryllidae	*Eugryllodesescalerae* (Bolívar, 1894)^1,^#	INV04014	IBIOR342-22	BOLD:AEP5287"	OR974785
Gryllidae	*Eugryllodesescalerae* (Bolívar, 1894)^1,^#	INV04015	IBIOR343-22	BOLD:AEP5287"	OR974822
Gryllidae	*Eugryllodesescalerae* (Bolívar, 1894)^1,^#	INV04046	IBIOR350-22	BOLD:AEP5287"	OR974421
Gryllidae	*Eumodicogryllusbordigalensis* (Latreille, 1804)	INV02554	IBIOR115-17	BOLD:ACU5350	OR974640
Gryllidae	*Eumodicogryllusbordigalensis* (Latreille, 1804)	INV02916	IBIOR149-19	BOLD:ACU5350	OR974751
Gryllidae	*Eumodicogryllusbordigalensis* (Latreille, 1804)	INV02918	IBIOR150-19	BOLD:ACU5350	OR974644
Gryllidae	*Eumodicogryllusbordigalensis* (Latreille, 1804)	INV02932	IBIOR151-19	BOLD:ACU5350	OR974663
Gryllidae	*Eumodicogryllusbordigalensis* (Latreille, 1804)	INV03553	IBIOR155-19	BOLD:ACU5350	OR974788
Gryllidae	*Eumodicogryllusbordigalensis* (Latreille, 1804)	INV04047	IBIOR162-19	BOLD:ACU5350	OR974683
Gryllidae	*Eumodicogryllusbordigalensis* (Latreille, 1804)	INV08718	IBIOR505-22	BOLD:ACU5350	OR974470
Gryllidae	*Eumodicogryllusbordigalensis* (Latreille, 1804)	INV08735	IBIOR506-22	BOLD:ACU5350	OR974662
Gryllidae	*Eumodicogryllusbordigalensis* (Latreille, 1804)	INV08804	IBIOR511-22	BOLD:ACU5350	OR974594
Gryllidae	*Eumodicogryllusbordigalensis* (Latreille, 1804)	INV10559	IBIOR530-22	BOLD:ACU5350	OR974603
Gryllidae	*Gryllomorphalongicauda* (Rambur, 1838)#	INV07535	IBIOR471-22	BOLD:AEP5097"	OR974478
Gryllidae	*Gryllomorphalongicauda* (Rambur, 1838)#	INV07551	IBIOR477-22	BOLD:AEP5097"	OR974602
Gryllidae	*Gryllomorphalongicauda* (Rambur, 1838)#	INV07552	IBIOR478-22	BOLD:AEP5097"	OR974744
Gryllidae	*Gryllomorphalongicauda* (Rambur, 1838)#	INV07767	IBIOR487-22	BOLD:AEP5096"	OR974694
Gryllidae	*Gryllomorphauclensis* Pantel, 1890#	INV03607	IBIOR312-22	BOLD:AEP5098"	OR974685
Gryllidae	*Gryllomorphauclensis* Pantel, 1890#	INV03710	IBIOR317-22	BOLD:AEP5098"	OR974418
Gryllidae	*Gryllomorphauclensis* Pantel, 1890#	INV04121	IBIOR361-22	BOLD:AEP5098"	OR974477
Gryllidae	*Gryllomorphauclensis* Pantel, 1890#	INV04122	IBIOR362-22	BOLD:AEP5098"	OR974555
Gryllidae	*Gryllomorphauclensis* Pantel, 1890#	INV07146	IBIOR460-22	BOLD:AEP5098"	OR974763
Gryllidae	*Gryllusbimaculatus* De Geer, 1773	INV02564	IBIOR122-17	BOLD:ABX6319	OR974451
Gryllidae	*Gryllusbimaculatus* De Geer, 1773	INV03744	IBIOR327-22	BOLD:ABX6319	OR974739
Gryllidae	*Gryllusbimaculatus* De Geer, 1773	INV06946	IBIOR451-22	BOLD:ABX6319	OR974680
Gryllidae	*Gryllusbimaculatus* De Geer, 1773	INV06963	IBIOR454-22	BOLD:ABX6319	OR974800
Gryllidae	*Gryllusbimaculatus* De Geer, 1773	INV10186	IBIOR517-22	BOLD:ABX6319	OR974595
Gryllidae	*Grylluscampestris* Linnaeus, 1758	INV02587	IBIOR177-22	BOLD:AAD6537	OR974715
Gryllidae	*Grylluscampestris* Linnaeus, 1758	INV03268	IBIOR152-19	BOLD:AAD6537	OR974660
Gryllidae	*Petaloptilafermini* Gorochov & Llorente del Moral, 2001^1,^#	INV03261	IBIOR301-22	BOLD:AEO7507	OR974593
Gryllidae	*Petaloptilafermini* Gorochov & Llorente del Moral, 2001^1,^#	INV03262	IBIOR302-22	BOLD:AEO7507	OR974460
Gryllidae	*Petaloptilafermini* Gorochov & Llorente del Moral, 2001^1,^#	INV03263	IBIOR303-22	BOLD:AEO7507	OR974498
Gryllidae	*Petaloptilafermini* Gorochov & Llorente del Moral, 2001^1,^#	INV03264	IBIOR304-22	BOLD:AEO7507	OR974557
Gryllidae	*Petaloptilafermini* Gorochov & Llorente del Moral, 2001^1,^#	INV03265	IBIOR305-22	BOLD:AEO7507	OR974537
Gryllidae	*Petaloptilagalaica* Domingo, 2021^1,^#	INV02494	IBIOR286-22	BOLD:AEO7506	OR974701
Gryllidae	*Petaloptilagalaica* Domingo, 2021^1,^#	INV02880	IBIOR287-22	BOLD:AEO7506	OR974670
Gryllidae	*Petaloptilagalaica* Domingo, 2021^1,^#	INV02881	IBIOR288-22	BOLD:AEO7506	OR974825
Gryllidae	*Petaloptilagalaica* Domingo, 2021^1,^#	INV02882	IBIOR289-22	BOLD:AEO7506	OR974487
Gryllidae	*Petaloptilagalaica* Domingo, 2021^1,^#	INV02890	IBIOR294-22	BOLD:AEO7506	OR974453
Gryllidae	*Petaloptilagalaica* Domingo, 2021^1,^#	INV02975	IBIOR298-22	BOLD:AEO7506	OR974652
Gryllidae	*Petaloptilagalaica* Domingo, 2021^1,^#	INV02976	IBIOR299-22	BOLD:AEO7506	OR974677
Gryllidae	*Petaloptilagalaica* Domingo, 2021^1,^#	INV02977	IBIOR300-22	BOLD:AEO7506	OR974706
Gryllidae	*Petaloptilagalaica* Domingo, 2021^1,^#	INV03671	IBIOR315-22	BOLD:AEO7506	OR974503
Gryllidae	*Petaloptilagalaica* Domingo, 2021^1,^#	INV03938	IBIOR330-22	BOLD:AEO7506	OR974702
Gryllidae	*Petaloptilagalaica* Domingo, 2021^1,^#	INV07145	IBIOR459-22	BOLD:AEO7506	OR974789
Gryllidae	*Petaloptilagalaica* Domingo, 2021^1,^#	INV09746	IBIOR584-22	BOLD:AEO7506	OR974629
Gryllidae	*Petaloptilagalaica* Domingo, 2021^1,^#	INV10284	IBIOR523-22	BOLD:AEO7506	OR974801
Gryllidae	*Sciobialusitanica* (Rambur, 1838)#	INV00645	IBIOR169-19	BOLD:ADV9764"	OR974617
Gryllidae	*Sciobialusitanica* (Rambur, 1838)#	INV05578	IBIOR229-22	BOLD:ADV9764"	OR974474
Gryllidae	*Sciobialusitanica* (Rambur, 1838)#	INV07347	IBIOR466-22	BOLD:ADV9764"	OR974653
Gryllidae	*Sciobialusitanica* (Rambur, 1838)#	INV08092	IBIOR243-22	BOLD:ADV9764"	OR974568
Gryllidae	*Sciobialusitanica* (Rambur, 1838)#	INV08476	IBIOR496-22	BOLD:ADV9764"	OR974558
Gryllidae	*Sciobialusitanica* (Rambur, 1838)#	INV08769	IBIOR508-22	BOLD:ADV9764"	OR974564
Gryllidae	*Svercuspalmetorum* (Krauss, 1902)#	INV08098	IBIOR249-22	BOLD:AEI3656	OR974553
Gryllidae	*Svercuspalmetorum* (Krauss, 1902)#	INV10188	IBIOR518-22	BOLD:AEI3656	OR974486
Gryllotalpidae	*Gryllotalpaafricana* Palisot de Beauvois, 1805	INV09085	IBIOR514-22	BOLD:AER6009	OR974770
Gryllotalpidae	*Gryllotalpavineae* Bennet-Clark, 1970#	INV02541	IBIOR106-17	BOLD:AEP5100"	OR974509
Gryllotalpidae	*Gryllotalpavineae* Bennet-Clark, 1970#	INV04101	IBIOR355-22	BOLD:AEP5099"	OR974817
Gryllotalpidae	*Gryllotalpavineae* Bennet-Clark, 1970#	INV04148	IBIOR364-22	BOLD:AEP5099"	OR974504
Mogoplistidae	*Arachnocephalusvestitus* Costa, 1855	INV05571	IBIOR222-22	BOLD:AER1092	OR974649
Mogoplistidae	*Paramogoplistesdentatus* Gorochov & Llorente del Moral, 2001^1^	INV07634	IBIOR483-22	BOLD:AEO7941"	OR974590
Mogoplistidae	*Paramogoplistesortini* Llucià Pomares, 2015^1^	INV07633	IBIOR482-22	BOLD:AEO7942"	OR974689
Mogoplistidae	*Paramogoplistesortini* Llucià Pomares, 2015^1^	INV07690	IBIOR486-22	BOLD:AEO7942"	OR974632
Mogoplistidae	*Pseudomogoplistesvicentae* Gorochov, 1996#	INV05575	IBIOR226-22	BOLD:AEI3508	OR974710
Mogoplistidae	*Pseudomogoplistesvicentae* Gorochov, 1996#	INV05576	IBIOR227-22	BOLD:AEI3508	OR974484
Oecanthidae	*Oecanthusdulcisonans* Gorochov, 1993#	INV05589	IBIOR240-22	BOLD:ACG7286	OR974741
Oecanthidae	*Oecanthuspellucens* (Scopoli, 1763)	INV00109	IBIOR270-22	BOLD:AAF4350	OR974458
Oecanthidae	*Oecanthuspellucens* (Scopoli, 1763)	INV00110	IBIOR044-17	BOLD:AAF4350	OR974627
Oecanthidae	*Oecanthuspellucens* (Scopoli, 1763)	INV01600	IBIOR143-19	BOLD:AAF4350	OR974588
Oecanthidae	*Oecanthuspellucens* (Scopoli, 1763)	INV03582	IBIOR156-19	BOLD:AAF4350	OR974779
Oecanthidae	*Oecanthuspellucens* (Scopoli, 1763)	INV03638	IBIOR158-19	BOLD:AAF4350	OR974621
Oecanthidae	*Oecanthuspellucens* (Scopoli, 1763)	INV07629	IBIOR481-22	BOLD:AAF4350	OR974560
Pamphagidae	*Acinipeignatii* Llorente del Moral & Presa, 1983^1,^#	INV05580	IBIOR231-22	BOLD:AER2325"	OR974628
Pamphagidae	*Acinipeignatii* Llorente del Moral & Presa, 1983^1,^#	INV05581	IBIOR232-22	BOLD:AER2325"	OR974475
Pamphagidae	*Eumigusayresi* Bolívar, 1912^1,^#	INV04381	IBIOR388-22	BOLD:AEP5224	OR974727
Pamphagidae	*Eumigusayresi* Bolívar, 1912^1,^#	INV04382	IBIOR389-22	BOLD:AEP5224	OR974687
Pamphagidae	*Eumigusayresi* Bolívar, 1912^1,^#	INV04384	IBIOR390-22	BOLD:AEP5224	OR974530
Pamphagidae	*Euryparyphesterrulentus* (Serville, 1838)^1,^#	INV00111	IBIOR045-17	BOLD:AEP5297"	OR974434
Pamphagidae	*Euryparyphesterrulentus* (Serville, 1838)^1,^#	INV00112	IBIOR046-17	BOLD:AEP5297"	OR974613
Pamphagidae	*Euryparyphesterrulentus* (Serville, 1838)^1,^#	INV02509	IBIOR074-17	BOLD:AEP5297"	OR974711
Pyrgomorphidae	*Pyrgomorphaconica* (Olivier, 1791)	INV02528	IBIOR093-17	BOLD:AEO6938	OR974695
Pyrgomorphidae	*Pyrgomorphaconica* (Olivier, 1791)	INV02586	IBIOR176-22	BOLD:AEO6938	OR974735
Tetrigidae	*Paratettixmeridionalis* (Rambur, 1838)	INV00113	IBIOR047-17	BOLD:AAP5007	OR974448
Tetrigidae	*Paratettixmeridionalis* (Rambur, 1838)	INV00114	IBIOR048-17	BOLD:AAP5007	OR974720
Tetrigidae	*Paratettixmeridionalis* (Rambur, 1838)	INV00169	IBIOR281-22	BOLD:AAP5007	OR974423
Tetrigidae	*Paratettixmeridionalis* (Rambur, 1838)	INV00487	IBIOR282-22	BOLD:AAP5007	OR974454
Tetrigidae	*Paratettixmeridionalis* (Rambur, 1838)	INV02534	IBIOR099-17	BOLD:AAP5007	OR974775
Tetrigidae	*Paratettixmeridionalis* (Rambur, 1838)	INV04327	IBIOR378-22	BOLD:AAP5007	OR974645
Tetrigidae	*Paratettixmeridionalis* (Rambur, 1838)	INV06862	IBIOR442-22	BOLD:AAP5007	OR974717
Tetrigidae	*Paratettixmeridionalis* (Rambur, 1838)	INV08590	IBIOR503-22	BOLD:AAP5007	OR974665
Tetrigidae	*Tetrixceperoi* (Bolívar, 1887)	INV03714	IBIOR321-22	BOLD:AAF1605	OR974591
Tetrigidae	*Tetrixceperoi* (Bolívar, 1887)	INV04328	IBIOR167-19	BOLD:AAF1605	OR974572
Tetrigidae	*Tetrixceperoi* (Bolívar, 1887)	INV04333	IBIOR168-19	BOLD:AAF1605	OR974427
Tetrigidae	*Tetrixdepressa* Brisout de Barneville, 1848	INV04324	IBIOR375-22	BOLD:AER2533	OR974747
Tetrigidae	*Tetrixdepressa* Brisout de Barneville, 1848	INV04335	IBIOR383-22	BOLD:AER2533	OR974463
Tetrigidae	*Tetrixnodulosa* (Fieber, 1853)#	INV02584	IBIOR139-17	BOLD:AEO6332"	OR974637
Tetrigidae	*Tetrixundulata* (Sowerby, 1806)	INV02886	IBIOR290-22	BOLD:ACU3727	OR974765
Tetrigidae	*Tetrixundulata* (Sowerby, 1806)	INV02887	IBIOR291-22	BOLD:ACU3727	OR974722
Tetrigidae	*Tetrixundulata* (Sowerby, 1806)	INV04322	IBIOR373-22	BOLD:ACU3727	OR974753
Tetrigidae	*Tetrixundulata* (Sowerby, 1806)	INV06353	IBIOR415-22	BOLD:ACU3727	OR974634
Tetrigidae	*Tetrixundulata* (Sowerby, 1806)	INV06531	IBIOR421-22	BOLD:ACU3727	OR974705
Tetrigidae	*Tetrixundulata* (Sowerby, 1806)	INV09776	IBIOR585-22	BOLD:ACU3727	OR974492
Tetrigidae	*Tetrixundulata* (Sowerby, 1806)	INV09777	IBIOR586-22	BOLD:ACU3727	OR974438
Tetrigidae	*Tetrixundulata* (Sowerby, 1806)	INV09862	IBIOR587-22	BOLD:ACU3727	OR974464
Tetrigidae	*Tetrixundulata* (Sowerby, 1806)	INV10036	IBIOR588-22	BOLD:ACU3727	OR974575
Tettigoniidae	*Antaxiusflorezi* Bolívar, 1900^1,^#	INV05548	IBIOR199-22	BOLD:AER0570	OR974719
Tettigoniidae	*Antaxiusflorezi* Bolívar, 1900^1,^#	INV05549	IBIOR200-22	BOLD:AER0570	OR974562
Tettigoniidae	*Antaxiusspinibrachius* (Fischer, 1853)#	INV01297	IBIOR284-22	BOLD:AER0568	OR974748
Tettigoniidae	*Antaxiusspinibrachius* (Fischer, 1853)#	INV03948	IBIOR332-22	BOLD:AER0568	OR974810
Tettigoniidae	*Antaxiusspinibrachius* (Fischer, 1853)#	INV03993	IBIOR336-22	BOLD:AER0568	OR974712
Tettigoniidae	*Antaxiusspinibrachius* (Fischer, 1853)#	INV03994	IBIOR337-22	BOLD:AER0568	OR974693
Tettigoniidae	*Antaxiusspinibrachius* (Fischer, 1853)#	INV04000	IBIOR339-22	BOLD:AER0568	OR974743
Tettigoniidae	*Antaxiusspinibrachius* (Fischer, 1853)#	INV04001	IBIOR340-22	BOLD:AER0568	OR974445
Tettigoniidae	*Antaxiusspinibrachius* (Fischer, 1853)#	INV04034	IBIOR347-22	BOLD:AER0567	OR974576
Tettigoniidae	*Antaxiusspinibrachius* (Fischer, 1853)#	INV04036	IBIOR348-22	BOLD:AER0567	OR974417
Tettigoniidae	*Antaxiusspinibrachius* (Fischer, 1853)#	INV04088	IBIOR352-22	BOLD:AER0567	OR974791
Tettigoniidae	*Antaxiusspinibrachius* (Fischer, 1853)#	INV05550	IBIOR201-22	BOLD:AER0569	OR974641
Tettigoniidae	*Antaxiusspinibrachius* (Fischer, 1853)#	INV05551	IBIOR202-22	BOLD:AER0569	OR974472
Tettigoniidae	*Antaxiusspinibrachius* (Fischer, 1853)#	INV07171	IBIOR567-22	BOLD:AER0569	OR974688
Tettigoniidae	*Antaxiusspinibrachius* (Fischer, 1853)#	INV07172	IBIOR568-22	BOLD:AER0569	OR974426
Tettigoniidae	*Antaxiusspinibrachius* (Fischer, 1853)#	INV07174	IBIOR569-22	BOLD:AER0567	OR974824
Tettigoniidae	*Conocephalusconocephalus* (Linnaeus, 1767)	INV05569	IBIOR220-22	BOLD:AEQ8460	OR974496
Tettigoniidae	*Conocephalusconocephalus* (Linnaeus, 1767)	INV05570	IBIOR221-22	BOLD:AEQ8460	OR974443
Tettigoniidae	*Conocephalusconocephalus* (Linnaeus, 1767)	INV06923	IBIOR445-22	BOLD:AEQ8460	OR974757
Tettigoniidae	*Conocephalusconocephalus* (Linnaeus, 1767)	INV06925	IBIOR446-22	BOLD:AEQ8460	OR974573
Tettigoniidae	*Conocephalusconocephalus* (Linnaeus, 1767)	INV06944	IBIOR449-22	BOLD:AEQ8460	OR974561
Tettigoniidae	*Conocephalusconocephalus* (Linnaeus, 1767)	INV06948	IBIOR453-22	BOLD:AEQ8460	OR974493
Tettigoniidae	*Conocephalusfuscus* (Fabricius, 1793)	INV05583	IBIOR234-22	BOLD:AEQ8462	OR974609
Tettigoniidae	*Conocephalusfuscus* (Fabricius, 1793)	INV06634	IBIOR435-22	BOLD:AEQ8462	OR974829
Tettigoniidae	*Conocephalusfuscus* (Fabricius, 1793)	INV09078	IBIOR513-22	BOLD:AEQ8462	OR974579
Tettigoniidae	*Cyrtaspisscutata* (Charpentier, 1825)#	INV07949	IBIOR493-22	BOLD:AEQ8625	OR974541
Tettigoniidae	*Cyrtaspisscutata* (Charpentier, 1825)#	INV08776	IBIOR510-22	BOLD:AEQ8625	OR974639
Tettigoniidae	*Cyrtaspisscutata* (Charpentier, 1825)#	INV09030	IBIOR512-22	BOLD:AEQ8625	OR974656
Tettigoniidae	*Decticusalbifrons* (Fabricius, 1775)#	INV00115	IBIOR049-17	BOLD:AEQ8654"	OR974549
Tettigoniidae	*Decticusalbifrons* (Fabricius, 1775)#	INV00116	IBIOR271-22	BOLD:AEQ8654"	OR974550
Tettigoniidae	*Decticusalbifrons* (Fabricius, 1775)#	INV02508	IBIOR073-17	BOLD:AEQ8654"	OR974446
Tettigoniidae	*Ephippigeridadiluta* (Bolívar, 1878)^1,^#	INV08107	IBIOR258-22	BOLD:AEP5780"	OR974778
Tettigoniidae	*Ephippigeridadiluta* (Bolívar, 1878)^1,^#	INV08108	IBIOR259-22	BOLD:AEP5780"	OR974678
Tettigoniidae	*Ephippigeridarosae* Barat & Correas, 2015^1,2,^#	INV08109	IBIOR260-22	BOLD:AEP5781"	OR974415
Tettigoniidae	*Incertanadecorata* (Fieber, 1853)#	INV02577	IBIOR133-17	BOLD:AEO9013	OR974700
Tettigoniidae	*Incertanadecorata* (Fieber, 1853)#	INV02578	IBIOR134-17	BOLD:AEO9013	OR974759
Tettigoniidae	*Leptophyespunctatissima* (Bosc, 1792)	INV05562	IBIOR213-22	BOLD:ADT5783	OR974467
Tettigoniidae	*Lluciapomaresiusanapaulae* (Schmidt, 2009)^1,2,^#	INV02573	IBIOR129-17	BOLD:AEO8290	OR974723
Tettigoniidae	*Lluciapomaresiusasturiensis* (Bolívar, 1898)^1,^#	INV03388	IBIOR307-22	BOLD:AEO6324"	OR974819
Tettigoniidae	*Lluciapomaresiusasturiensis* (Bolívar, 1898)^1,^#	INV03952	IBIOR333-22	BOLD:AEO6324"	OR974672
Tettigoniidae	*Lluciapomaresiusasturiensis* (Bolívar, 1898)^1,^#	INV05560	IBIOR211-22	BOLD:AEO8296	OR974648
Tettigoniidae	*Lluciapomaresiusasturiensis* (Bolívar, 1898)^1,^#	INV05561	IBIOR212-22	BOLD:AEO8296	OR974697
Tettigoniidae	*Lluciapomaresiusstalii* (Bolívar, 1877)^1,^#	INV08100	IBIOR251-22	BOLD:AEO8295	OR974783
Tettigoniidae	*Meconemathalassinum* (De Geer, 1773)	INV03732	IBIOR159-19	BOLD:AAK7060	OR974440
Tettigoniidae	*Meconemathalassinum* (De Geer, 1773)	INV03944	IBIOR160-19	BOLD:AAK7060	OR974750
Tettigoniidae	*Meconemathalassinum* (De Geer, 1773)	INV04094	IBIOR165-19	BOLD:AAK7060	OR974507
Tettigoniidae	*Metriopteraambigua* Pfau, 1986^1,^#	INV02600	IBIOR190-22	BOLD:AEO8174	OR974461
Tettigoniidae	*Metriopteraambigua* Pfau, 1986^1,^#	INV05542	IBIOR193-22	BOLD:AEO8174	OR974767
Tettigoniidae	*Neocallicraniabarrosi* Barat, 2013^1,2,^#	INV08101	IBIOR252-22	BOLD:AEO7827"	OR974485
Tettigoniidae	*Neocallicraniabarrosi* Barat, 2013^1,2,^#	INV08102	IBIOR253-22	BOLD:AEO7827"	OR974563
Tettigoniidae	*Neocallicranialusitanica* (Aires & Menano, 1916)^1,^#	INV08110	IBIOR261-22	BOLD:AEO7822	OR974578
Tettigoniidae	*Neocallicranialusitanica* (Aires & Menano, 1916)^1,^#	INV08111	IBIOR262-22	BOLD:AEO7822	OR974435
Tettigoniidae	*Neocallicranialusitanica* (Aires & Menano, 1916)^1,^#	INV08112	IBIOR263-22	BOLD:AEO7826"	OR974827
Tettigoniidae	*Neocallicraniamiegii* (Bolívar, 1873)^1,^#	INV08103	IBIOR254-22	BOLD:AEO7823	OR974490
Tettigoniidae	*Neocallicraniamiegii* (Bolívar, 1873)^1,^#	INV08104	IBIOR255-22	BOLD:AEO7823	OR974513
Tettigoniidae	*Neocallicraniaselligerameridionalis* (Pfau, 1996)^1,^#	INV02574	IBIOR130-17	BOLD:AEO7820"	OR974631
Tettigoniidae	*Neocallicraniaselligerameridionalis* (Pfau, 1996)^1,^#	INV04091	IBIOR354-22	BOLD:AEO7825	OR974528
Tettigoniidae	*Neocallicraniaselligerameridionalis* (Pfau, 1996)^1,^#	INV07436	IBIOR470-22	BOLD:AEO7825	OR974462
Tettigoniidae	*Neocallicraniaselligeraselligera* (Charpentier, 1825)^1,^#	INV06585	IBIOR429-22	BOLD:AEO7824"	OR974724
Tettigoniidae	*Neocallicraniaserrata* (Bolívar, 1885)^1,2,^#	INV08105	IBIOR256-22	BOLD:AEO7821"	OR974605
Tettigoniidae	*Neocallicraniaserrata* (Bolívar, 1885)^1,2,^#	INV08106	IBIOR257-22	BOLD:AEO7821"	OR974589
Tettigoniidae	*Odonturaglabricauda* (Charpentier, 1825)#	INV00117	IBIOR050-17	BOLD:AEO7751	OR974651
Tettigoniidae	*Odonturaglabricauda* (Charpentier, 1825)#	INV02515	IBIOR080-17	BOLD:AEO7751	OR974587
Tettigoniidae	*Odonturaglabricauda* (Charpentier, 1825)#	INV06518	IBIOR419-22	BOLD:AEO7751	OR974459
Tettigoniidae	*Odonturaglabricauda* (Charpentier, 1825)#	INV06520	IBIOR420-22	BOLD:AEO7751	OR974804
Tettigoniidae	*Odonturaglabricauda* (Charpentier, 1825)#	INV08771	IBIOR509-22	BOLD:AEO7751	OR974664
Tettigoniidae	*Odonturamacphersoni* Morales-Agacino, 1943^1,^#	INV05587	IBIOR238-22	BOLD:AEO7752	OR974608
Tettigoniidae	*Odonturamacphersoni* Morales-Agacino, 1943^1,^#	INV05588	IBIOR239-22	BOLD:AEO7752	OR974456
Tettigoniidae	*Phaneropteranana* Fieber, 1853	INV02559	IBIOR117-17	BOLD:AEO7449	OR974643
Tettigoniidae	*Phaneropteranana* Fieber, 1853	INV03968	IBIOR334-22	BOLD:AEO7449	OR974614
Tettigoniidae	*Phaneropteranana* Fieber, 1853	INV03978	IBIOR335-22	BOLD:AEO7449	OR974650
Tettigoniidae	*Phaneropteranana* Fieber, 1853	INV04008	IBIOR341-22	BOLD:AEO7449	OR974667
Tettigoniidae	*Phaneropteranana* Fieber, 1853	INV04019	IBIOR344-22	BOLD:AEO7449	OR974798
Tettigoniidae	*Phaneropteranana* Fieber, 1853	INV04021	IBIOR345-22	BOLD:AEO7449	OR974799
Tettigoniidae	*Phaneropteranana* Fieber, 1853	INV04042	IBIOR349-22	BOLD:AEO7449	OR974444
Tettigoniidae	*Phaneropteranana* Fieber, 1853	INV06063	IBIOR408-22	BOLD:AEO7449	OR974764
Tettigoniidae	*Phaneropteranana* Fieber, 1853	INV06087	IBIOR412-22	BOLD:AEO7449	OR974416
Tettigoniidae	*Phaneropteranana* Fieber, 1853	INV06558	IBIOR424-22	BOLD:AEO7449	OR974510
Tettigoniidae	*Phaneropteranana* Fieber, 1853	INV06560	IBIOR425-22	BOLD:AEO7449	OR974758
Tettigoniidae	*Phaneropteranana* Fieber, 1853	INV06629	IBIOR433-22	BOLD:AEO7449	OR974655
Tettigoniidae	*Phaneropteranana* Fieber, 1853	INV09088	IBIOR515-22	BOLD:AEO7449	OR974469
Tettigoniidae	*Phaneropteranana* Fieber, 1853	INV10234	IBIOR520-22	BOLD:AEO7449	OR974481
Tettigoniidae	*Phaneropteranana* Fieber, 1853	INV10271	IBIOR522-22	BOLD:AEO7449	OR974586
Tettigoniidae	*Phaneropteranana* Fieber, 1853	INV10339	IBIOR528-22	BOLD:AEO7449	OR974813
Tettigoniidae	*Phaneropteranana* Fieber, 1853	INV11453	IBIOR555-22	BOLD:AEO7449	OR974449
Tettigoniidae	*Phaneropterasparsa* Stål, 1857#	INV06918	IBIOR444-22	BOLD:AEO7448"	OR974512
Tettigoniidae	*Phaneropterasparsa* Stål, 1857#	INV06926	IBIOR447-22	BOLD:AEO7448"	OR974518
Tettigoniidae	*Phaneropterasparsa* Stål, 1857#	INV07540	IBIOR472-22	BOLD:AEO7448"	OR974619
Tettigoniidae	*Phaneropterasparsa* Stål, 1857#	INV07543	IBIOR474-22	BOLD:AEO7448"	OR974771
Tettigoniidae	*Platycleisaffinis* Fieber, 1853	INV00122	IBIOR053-17	BOLD:AAH9443	OR974752
Tettigoniidae	*Platycleisaffinis* Fieber, 1853	INV02516	IBIOR081-17	BOLD:AAH9443	OR974816
Tettigoniidae	*Platycleisalbopunctata* (Goeze, 1778)	INV03998	IBIOR338-22	BOLD:AEO6325	OR974784
Tettigoniidae	*Platycleisalbopunctata* (Goeze, 1778)	INV05544	IBIOR195-22	BOLD:AEO6325	OR974442
Tettigoniidae	*Platycleisalbopunctata* (Goeze, 1778)	INV06511	IBIOR417-22	BOLD:AEO6325	OR974815
Tettigoniidae	*Platycleisalbopunctata* (Goeze, 1778)	INV07110	IBIOR458-22	BOLD:AEO6325	OR974626
Tettigoniidae	*Platycleisintermedia* (Serville, 1838)	INV02518	IBIOR083-17	BOLD:AAE4790	OR974615
Tettigoniidae	*Platycleisintermedia* (Serville, 1838)	INV10586	IBIOR532-22	BOLD:AAE4790	OR974728
Tettigoniidae	*Platycleissabulosa* Azam, 1901#	INV00131	IBIOR058-17	BOLD:AEO6325	OR974479
Tettigoniidae	*Platycleissabulosa* Azam, 1901#	INV00132	IBIOR059-17	BOLD:AEO6325	OR974756
Tettigoniidae	*Platycleissabulosa* Azam, 1901#	INV00133	IBIOR060-17	BOLD:AEO6325	OR974450
Tettigoniidae	*Platycleissabulosa* Azam, 1901#	INV00134	IBIOR061-17	BOLD:AEO6325	OR974690
Tettigoniidae	*Platycleissabulosa* Azam, 1901#	INV02572	IBIOR128-17	BOLD:AEO6325	OR974581
Tettigoniidae	*Platycleissabulosa* Azam, 1901#	INV05439	IBIOR399-22	BOLD:AAL4618	OR974658
Tettigoniidae	*Platycleissabulosa* Azam, 1901#	INV05515	IBIOR403-22	BOLD:AEO6325	OR974429
Tettigoniidae	*Platycleissabulosa* Azam, 1901#	INV05516	IBIOR404-22	BOLD:AAL4618	OR974716
Tettigoniidae	*Platycleissabulosa* Azam, 1901#	INV07352	IBIOR468-22	BOLD:AEO6325	OR974679
Tettigoniidae	*Platycleissabulosa* Azam, 1901#	INV07353	IBIOR469-22	BOLD:AAL4618	OR974671
Tettigoniidae	*Platycleissabulosa* Azam, 1901#	INV10326	IBIOR527-22	BOLD:AEO6325	OR974468
Tettigoniidae	*Platycleissabulosa* Azam, 1901#	INV10593	IBIOR534-22	BOLD:AAL4618	OR974814
Tettigoniidae	*Platycleissabulosa* Azam, 1901#	INV11434	IBIOR551-22	BOLD:AEO6325	OR974630
Tettigoniidae	*Platycleissabulosa* Azam, 1901#	INV11452	IBIOR554-22	BOLD:AEO6325	OR974596
Tettigoniidae	*Platystolusmartinezii* (Bolívar, 1873)^1,^#	INV02526	IBIOR091-17	BOLD:AEO7253	OR974818
Tettigoniidae	*Platystolusmartinezii* (Bolívar, 1873)^1,^#	INV02527	IBIOR092-17	BOLD:AEO7253	OR974545
Tettigoniidae	*Pterolepisgrallata* (Pantel, 1886)#	INV06068	IBIOR410-22	BOLD:AEO7102"	OR974774
Tettigoniidae	*Pterolepisgrallata* (Pantel, 1886)#	INV06069	IBIOR411-22	BOLD:AEO7102"	OR974761
Tettigoniidae	*Pterolepisgrallata* (Pantel, 1886)#	INV06609	IBIOR431-22	BOLD:AEO7102"	OR974483
Tettigoniidae	*Pterolepisgrallata* (Pantel, 1886)#	INV06631	IBIOR434-22	BOLD:AEO7102"	OR974566
Tettigoniidae	*Pterolepisgrallata* (Pantel, 1886)#	INV07175	IBIOR570-22	BOLD:AEO7102"	OR974830
Tettigoniidae	*Pterolepisgrallata* (Pantel, 1886)#	INV07351	IBIOR467-22	BOLD:AEO7102"	OR974809
Tettigoniidae	*Pterolepislusitanica* (Bolívar, 1900)^1,2,^#	INV05574	IBIOR225-22	BOLD:AEO7101	OR974551
Tettigoniidae	*Pterolepislusitanica* (Bolívar, 1900)^1,2,^#	INV07563	IBIOR479-22	BOLD:AEO7101	OR974437
Tettigoniidae	*Pterolepisspoliata* Rambur, 1838^1,^#	INV06945	IBIOR450-22	BOLD:AEO7101	OR974831
Tettigoniidae	*Pterolepisspoliata* Rambur, 1838^1,^#	INV08090	IBIOR241-22	BOLD:AEO7101	OR974424
Tettigoniidae	*Pycnogastercucullatus* (Charpentier, 1825)^1,2,^#	INV02590	IBIOR180-22	BOLD:AEO6978"	OR974521
Tettigoniidae	*Pycnogastercucullatus* (Charpentier, 1825)^1,2,^#	INV05577	IBIOR228-22	BOLD:AEO6980"	OR974622
Tettigoniidae	*Pycnogasterjugicola* Graells, 1851^1,^#	INV05563	IBIOR214-22	BOLD:AEO6979"	OR974704
Tettigoniidae	*Ruspolianitidula* (Scopoli, 1786)	INV02561	IBIOR119-17	BOLD:AAJ7497	OR974654
Tettigoniidae	*Ruspolianitidula* (Scopoli, 1786)	INV04086	IBIOR163-19	BOLD:AAJ7497	OR974782
Tettigoniidae	*Ruspolianitidula* (Scopoli, 1786)	INV04089	IBIOR164-19	BOLD:AAJ7497	OR974529
Tettigoniidae	*Steropleuruspseudolus* (Bolívar, 1878)^1,^#	INV02592	IBIOR182-22	BOLD:AEO6528"	OR974515
Tettigoniidae	*Steropleuruspseudolus* (Bolívar, 1878)^1,^#	INV02593	IBIOR183-22	BOLD:AEO6528"	OR974620
Tettigoniidae	*Tessellanatessellata* (Charpentier, 1825)	INV00136	IBIOR062-17	BOLD:AER1298	OR974473
Tettigoniidae	*Tessellanatessellata* (Charpentier, 1825)	INV00137	IBIOR063-17	BOLD:AAF2021	OR974526
Tettigoniidae	*Tessellanatessellata* (Charpentier, 1825)	INV03387	IBIOR154-19	BOLD:AAF2021	OR974616
Tettigoniidae	*Tessellanatessellata* (Charpentier, 1825)	INV06544	IBIOR422-22	BOLD:AER1298	OR974556
Tettigoniidae	*Tessellanatessellata* (Charpentier, 1825)	INV06545	IBIOR423-22	BOLD:AAF2021	OR974522
Tettigoniidae	*Tessellanatessellata* (Charpentier, 1825)	INV09723	IBIOR583-22	BOLD:AER6209"	OR974718
Tettigoniidae	*Tettigoniaviridissima* (Linnaeus, 1758)	INV02524	IBIOR089-17	BOLD:AAF1423	OR974547
Tettigoniidae	*Tettigoniaviridissima* (Linnaeus, 1758)	INV02525	IBIOR090-17	BOLD:AAF1423	OR974531
Tettigoniidae	*Tettigoniaviridissima* (Linnaeus, 1758)	INV05451	IBIOR401-22	BOLD:AAF1423	OR974520
Tettigoniidae	*Thyreonotusbidens* (Bolívar, 1887)^1,^#	INV02581	IBIOR137-17	BOLD:AER1533"	OR974766
Tettigoniidae	*Thyreonotusbidens* (Bolívar, 1887)^1,^#	INV04118	IBIOR359-22	BOLD:AER1532	OR974598
Tettigoniidae	*Thyreonotusbidens* (Bolívar, 1887)^1,^#	INV05307	IBIOR396-22	BOLD:AER1532	OR974597
Tettigoniidae	*Thyreonotusbidens* (Bolívar, 1887)^1,^#	INV06570	IBIOR427-22	BOLD:AER1532	OR974439
Tettigoniidae	*Thyreonotusbidens* (Bolívar, 1887)^1,^#	INV07109	IBIOR457-22	BOLD:AER1532	OR974499
Tettigoniidae	*Thyreonotusbidens* (Bolívar, 1887)^1,^#	INV07232	IBIOR462-22	BOLD:AER1532	OR974725
Tettigoniidae	*Thyreonotusbidens* (Bolívar, 1887)^1,^#	INV07796	IBIOR490-22	BOLD:AER1531"	OR974500
Tettigoniidae	*Thyreonotusbidens* (Bolívar, 1887)^1,^#	INV07805	IBIOR491-22	BOLD:AER1533"	OR974471
Tettigoniidae	*Tylopsislilifolia* (Fabricius, 1793)#	INV00140	IBIOR064-17	BOLD:AER2541	OR974552
Tettigoniidae	*Tylopsislilifolia* (Fabricius, 1793)#	INV00141	IBIOR065-17	BOLD:AER2541	OR974797
Tettigoniidae	*Tylopsislilifolia* (Fabricius, 1793)#	INV02520	IBIOR085-17	BOLD:AER2541	OR974792
Tettigoniidae	*Tylopsislilifolia* (Fabricius, 1793)#	INV02521	IBIOR086-17	BOLD:AER2541	OR974422
Tettigoniidae	*Tylopsislilifolia* (Fabricius, 1793)#	INV10591	IBIOR533-22	BOLD:AER2541	OR974502
Tridactylidae	*Xyaiberica* Günther, 1990^1,^#	INV02567	IBIOR124-17	BOLD:AER2238	OR974754
Trigonidiidae	*Natulaaverni* (Costa, 1855)#	INV02565	IBIOR123-17	BOLD:ACP3886	OR974642
Trigonidiidae	*Natulaaverni* (Costa, 1855)#	INV02582	IBIOR138-17	BOLD:ACP3886	OR974795
Trigonidiidae	*Nemobiussylvestris* (Bosc, 1792)	INV02258	IBIOR144-19	BOLD:AAC3438	OR974524
Trigonidiidae	*Nemobiussylvestris* (Bosc, 1792)	INV02570	IBIOR126-17	BOLD:AER5488"	OR974523
Trigonidiidae	*Nemobiussylvestris* (Bosc, 1792)	INV02571	IBIOR127-17	BOLD:AER5488"	OR974691
Trigonidiidae	*Nemobiussylvestris* (Bosc, 1792)	INV03615	IBIOR157-19	BOLD:AAC3438	OR974826
Trigonidiidae	*Nemobiussylvestris* (Bosc, 1792)	INV03758	IBIOR328-22	BOLD:AAC3438	OR974675
Trigonidiidae	*Nemobiussylvestris* (Bosc, 1792)	INV03759	IBIOR329-22	BOLD:AAC3438	OR974769
Trigonidiidae	*Nemobiussylvestris* (Bosc, 1792)	INV07640	IBIOR484-22	BOLD:AEO7832"	OR974684
Trigonidiidae	*Nemobiussylvestris* (Bosc, 1792)	INV07769	IBIOR489-22	BOLD:AEO7832"	OR974746
Trigonidiidae	*Nemobiussylvestris* (Bosc, 1792)	INV08603	IBIOR504-22	BOLD:AER5488"	OR974466
Trigonidiidae	*Pteronemobiuslineolatus* (Brullé, 1835)#	INV03739	IBIOR325-22	BOLD:AEO6935	OR974731
Trigonidiidae	*Trigonidiumcicindeloides* Rambur, 1838	INV02529	IBIOR094-17	BOLD:ACY3774	OR974533
Trigonidiidae	*Trigonidiumcicindeloides* Rambur, 1838	INV02530	IBIOR095-17	BOLD:ACY3774	OR974420
Trigonidiidae	*Trigonidiumcicindeloides* Rambur, 1838	INV07034	IBIOR456-22	BOLD:ACY3774	OR974412
Trigonidiidae	*Trigonidiumcicindeloides* Rambur, 1838	INV10193	IBIOR519-22	BOLD:ACY3774	OR974786

**Table 2.** Number of specimens and species collected per Portuguese district.

**District**	**Number of specimens**	**Number of species**
Beja	108	44
Bragança	95	40
Castelo Branco	14	9
Coimbra	4	4
Évora	1	1
Faro	21	13
Guarda	36	23
Leiria	11	6
Lisboa	6	4
Portalegre	1	1
Porto	7	7
Santarém	9	6
Setúbal	68	45
Viana do Castelo	17	9
Vila Real	22	14
Total	420	119
